# Striatal miR-183-5p inhibits methamphetamine-induced locomotion by regulating glucocorticoid receptor signaling

**DOI:** 10.3389/fphar.2022.997701

**Published:** 2022-09-26

**Authors:** Sang-Hoon Song, Won-Jun Jang, Eun Young Jang, Oc-Hee Kim, Haesoo Kim, Taekwon Son, Dong-Young Choi, Sooyeun Lee, Chul-Ho Jeong

**Affiliations:** ^1^ College of Pharmacy, Keimyung University, Daegu, South Korea; ^2^ Pharmacology and Drug Abuse Research Group, Korea Institute of Toxicology, Daejeon, South Korea; ^3^ Korea Brain Bank, Korea Brain Research Institute, Daegu, South Korea; ^4^ College of Pharmacy, Yeungnam University, Gyeongsan, South Korea

**Keywords:** methamphetamine, RNA sequencing, microRNA, self-administration, locomotor activity, glucocorticoid receptor

## Abstract

MicroRNA (miRNA)-mediated striatal gene regulation may play an important role in methamphetamine (METH) addiction. This study aimed to identify changes in novel miRNAs and their target genes during METH self-administration and investigate their roles in METH-induced locomotion. RNA sequencing analysis revealed that mir-183-5p was upregulated in the striatum of METH self-administered rats, and target gene prediction revealed that the glucocorticoid receptor (GR) gene, *Nr3c1*, was a potential target gene for mir-183-5p. We confirmed that single and repeated METH administrations increased METH-induced locomotion and plasma corticosterone levels in rats. Additionally, increased miR-185-5p expression and decreased GR gene expression were observed only in the repeated-METH-injection group but not in the single-injection group. We then investigated the effects of miR-183-5p on METH-induced locomotion using a miR-183-5p mimic and inhibitor. Injection of a mir-183-5p mimic in the striatum of rats attenuated METH-induced locomotion, whereas injection of a miR-183-5p inhibitor enhanced the locomotor activity in METH-administered rats. Furthermore, the miR-183-5p mimic reduced the phosphorylation of tyrosine hydroxylase (TH) whereas the inhibitor increased it. Taken together, these results indicate that repeated METH injections increase striatal miR-183-5p expression and regulate METH-induced locomotion by regulating GR expression in rats, thereby suggesting a potential role of miR-183-5p as a novel regulator of METH-induced locomotion.

## Introduction

Methamphetamine (METH) is a strong psychostimulant that causes behavioral and cognitive deficits. Chronic administration of psychostimulants, such as METH, leads to physical and psychological dependence that is conceptualized as a cycle comprising rewarding effect, withdrawal effect, craving, and relapse to drug seeking behavior (Goldstein and Volkow, 2002; Cami and Farre, 2003). Behavioral sensitization by repeated drug administration is considered a primary stage for drug addiction, craving, and certain aspects of drug-induced psychosis (Robinson and Becker, 1986). METH enhances behavioral change by acting on the mesolimbic dopaminergic system, which is related to reward and motivation through dopamine (DA) regulation in the synaptic cleft (Di Chiara, 2002; Yao et al., 2010). Although many studies have investigated the molecular changes in the signal transduction of addiction-related genes and pathways in the brain, the exact mechanism underlying METH addiction remains unclear. Therefore, continuous attempts are still needed to identify the mechanism and diagnostic markers of METH addiction using animal models for METH reward processes, and many studies are currently in progress (Song et al., 2018; Kim M. et al., 2019; Jang et al., 2020; Kim et al., 2020). The rat model of self-administration and locomotion has been proposed as an animal model to investigate the effects of drug abuse (Steketee and Kalivas, 2011; Jing et al., 2014; Perrine et al., 2015).

Drug abuse induces dysregulation of the stress neurobiological system (Edwards and Koob, 2010). Glucocorticoids (GCs) are major stress hormones of the hypothalamic-pituitary-adrenal (HPA) axis, which act on glucocorticoid receptors (GR) or mineralocorticoid receptors (MR). Upon binding to GCs, GR acts as a transcription factor (TF) that regulates the transcription of various target genes by forming complexes with other TFs (Timmermans et al., 2019). Interestingly, repeated exposure to cocaine, followed by withdrawal, has been shown to activate the neuroendocrine stress response in rats (Avila et al., 2003). Additionally, GCs are involved in METH-induced hyperactivity in mice (Ago et al., 2009), indicating that changes in the HPA axis function are related to altered stress-related behaviors and might contribute to addictive processes such as relapse (Zuloaga et al., 2015). A recent study has shown that GR knockout in the central nervous system (CNS) reduces cocaine-seeking behavior, suggesting that GR is a potential target to reduce cocaine abuse (Deroche-Gamonet et al., 2003). Moreover, the administration of mifepristone, a GR antagonist, decreases cocaine and amphetamine self-administration (Fiancette et al., 2010; Stairs et al., 2011), implying the role of GR in drug addiction. Chronic administration of METH and amphetamine decreases hippocampal GR and MR mRNA levels in rats (Kabbaj et al., 2003). Additionally, *NR3C1*, the gene name of GR, is decreased in the blood of cocaine users (Schote et al., 2019).

MicroRNAs (miRNAs) have been shown to play a crucial role in drug addiction. The striatal miR-8 family (miR-141, miR-200a, miR-200b, miR-200c, and miR-429) play a role in the cocaine-mediated maintenance of synaptic plasticity (Eipper-Mains et al., 2011). Overexpression of miR-124 inhibits cocaine-mediated microglial activation (Periyasamy et al., 2018). Additionally, miR-146a plays a role as a negative regulator of morphine analgesic tolerance (Wang et al., 2020). Previous study has confirmed the role of miRNAs in METH addiction, such as miR-124 and miR-181a which were identified through transcriptome analysis of the ventral tegmental area of METH self-administered rats (Bosch et al., 2015). Moreover, several miRNAs related to METH addiction in the nucleus accumbens (NAc) were identified following repeated METH administration in rats (Sim et al., 2017).

In this study, we performed small and total RNA sequencing to illustrate the transcriptome profiling in METH self-administered rats. We found that miR-183-5p was upregulated in the rat striatum following METH self-administration and confirmed that its target gene, *Nr3c1* (GR), was downregulated under the same experimental conditions. Overexpression of miR-183-5p in the rat striatum attenuated METH-induced locomotion, while miR-183-5p inhibition enhanced METH-induced locomotion. Our results demonstrated that the regulation of GR through miR-183-5p in the striatum is a novel mechanism that modulates METH-induced locomotion, suggesting the potential role of miR-183-5p in the regulation of the neuroendocrine system during METH addiction.

## Materials and methods

### Animals

Male Sprague-Dawley rats (Hyo-Chang Science, Daegu, Korea), weighing 200–220 g, were housed individually in cages in the laboratory animal facility under controlled temperature and humidity with a 12 h light-and-dark cycle. The experimental procedure for self-administration was approved by the Institutional Animal Care and Use Committee of the Korea Institute of Toxicology (approval no. KIT-1702-0076), and locomotion experiments were approved by the Institutional Animal Care and Use Committee of Keimyung University (approval no. KM 2021-009).

### Chemicals and reagents

All cell culture reagents were purchased from HyClone Laboratories (Logan, UT, USA). (+)-methamphetamine hydrochloride, 4′6-diamidino-2-phenylindole (DAPI), and dimethyl sulfoxide (DMSO) were purchased from Sigma-Aldrich (St. Louis, MO, USA). Dexamethasone and RU-486 were purchased from MCE Med Chem Express (Monmouth Junction, NJ, USA). Primary antibodies against p-TH (S40, #2791, AB_2201522), p-GR (S211, #4161, AB_2155797), and β-actin (#4970, AB_2223172), and secondary antibodies were purchased from Cell Signaling Technology (Danvers, MA, USA). Primary antibodies against TH (sc-25269, AB_628422), GR (sc-393232, AB_2687823), and GAPDH (sc-365062, AB_10847862) were purchased from Santa Cruz Biotechnology (Santa Cruz, CA, USA). MiR-183-5p mimic, inhibitor, and negative controls (NCs) were purchased from Qiagen (Hilden, Germany) and the sequence information is shown in Supplementary Table S1. The primer for miR-183-5p was purchased from Qiagen (Hilden, Germany). The primers for *U6*, *RNU48*, *5S*, *Nr3c1*, and *β-actin* were designed by Primer3Plus, and purchased from Bioneer (Daejeon, Republic of Korea). The sequence information is shown in Supplementary Table S2.

### Stereotactic surgery and intrastriatal injection

Stereotactic surgery and intrastriatal injection were performed as described previously (Hollander et al., 2010). For the intra-striatal administration of miR-183-5p mimics and inhibitors, chronic indwelling intracerebral cannulas were implanted above the dorsal striatum. Briefly, rats were anesthetized by inhalation of 2–3% isoflurane in oxygen and positioned in a stereotaxic frame (RWD life science, CA, USA). Bilateral stainless-steel guide cannula (23 gauge, 12 mm in length) was implanted 2 mm above the most dorsal injection site in the dorsal striatum according to the following stereotaxic coordinates: AP:1 mm from bregma; ML: ± 2.5 mm from midline; DV: -4, -5 mm from the dura. Two stainless-steel skull screws and a dental acrylic held the cannula in place. The miR-183-5p mimic and inhibitor were delivered on two consecutive days. On each day, rats were anesthetized after daily METH injection and received a total of two injections (1 μl per injection; 50 μM concentration) into each side of the dorsal striatum (a total of four striatal injection per rat per day). A stainless-steel injector (32 gauge, 16 mm in length) was inserted into the injection site. The molecule was delivered for more than 60 s. After infusion, the injector was left in place for 2 min. The injector was then raised by 1 mm to the next injection site, and the injection procedure was repeated. After the final injection, the dummy cannula was reinserted into the cannula, and a dust cap was screwed for protection.

### Self-administration

Self-administration of saline or METH was performed as previously described (Kim S. et al., 2019). To train the rats to perform lever presses (1 h/day) before drug self-administration, food training was conducted using a 45 mg food pellet. Only rats that acquired 80 food pellets per day for three consecutive days were selected for catheter surgery. A chronic indwelling jugular catheter (inner diameter, 0.02”; outer diameter, 0.03”; Dow Corning, Midland, MI, USA) was implanted in the right jugular vein under pentobarbital anesthesia (50 mg/kg, intraperitoneal). The catheter was secured to the jugular vein using a mersilene surgical mesh (Ethicon Inc., Raritan, NJ, USA) and exteriorized via a skin incision on the animal’s back through a 22-gauge stainless-steel cannula (P1 Technologies, Roanoke, VA, USA) fixed to the head assembly with dental cement and secured with a Prolene surgical mesh (Ethicon Inc.). After at least 5 days of recovery, the rats were allowed to self-administer METH (0.05 mg/kg/infusion) dissolved in saline in the form of METH hydrochloride (*n* = 24) or only saline as a control (*n* = 8) during a 2 h session under a fixed-ratio 1 (FR-1) 20 s timeout reinforcement schedule lasting for 16 days. Finally, rats showing a stable response (less than 20% variation) to the lever press during the last 3 days (*n* = 4 for both METH and saline self-administration) were chosen for plasma sampling. The rats in the saline self-administration group were treated using the same procedures, including catheter surgery, as the METH self-administration group. Catheter patency was maintained by flushing with 0.2 ml of saline containing heparin (30 IU/ml) and gentamycin sulfate (0.33 mg/ml) during the experimental period. Neither catheter patency failure nor health-related issues were observed throughout the self-administered experiment. Rats were anesthetized with a mixture of ketamine (72 mg/kg) and xylazine (6 mg/kg) *via* intraperitoneal injection and sacrificed to obtain brain samples.

### Locomotor activity

The locomotor activity test was performed on the last day of the saline or METH injections. Briefly, the experimental schedule was a 2-days pre-test to measure baseline locomotor activity and was randomly allocated to an indicated group. After the pre-test, the rats were habituated to the testing boxes before the experiment by being handled in the colony room for 20 min daily. On the day of the test, each rat was placed in a black Plexiglas square box (50 cm × 50 cm × 50 cm), and after an adaptation period of 15 min, saline, or METH (1 mg/kg) was injected intraperitoneally. Locomotor activity was measured and displayed as the total distance (cm) travelled during the 60 min period following saline or METH injections. The apparatus was cleaned using 70% alcohol and dried after each session. The distance was measured by an automated video tracking system (Smart Video Tracking System- SMART 3.0, Panlab, Barcelona, Spain). The video files were analyzed using DigBehv analysis software to calculate the motion tracking values, duration by movement speed, and total distance travelled.

### RNA extraction

Total RNA was extracted from HEK293 cells and striatal samples using the conventional method (TRIzol^®^ reagent; Invitrogen, Carlsbad, CA, USA). RNA quality and concentration were assessed using a filter-based multi-mode microplate reader (FLUOstar Omega, BMG Labtech, Ortenberg, Germany).

### Quantitative real-time polymerase chain reaction

For miRNAs, cDNA was synthesized from 50 ng of total RNA using miRCURY LNA RT Kit (Qiagen, Hilden, Germany), and miRNA expression levels were determined by qRT-PCR analysis using the miRCURY LNA miRNA PCR Assay kit (Qiagen, Hilden, Germany). All reactions were performed in triplicate and the results were normalized with *U6* or other reference genes *RNU48*, and *5S*. Amplification was performed using a reaction cycle at 95°C for 2 min, 95°C for 10 s, and 56°C for 1 min. For *Nr3c1* expression, qRT-PCR analysis was performed using TB Green^®^ Premix Ex Taq^™^ (Takara Bio Inc., Kusatsu, Shiga, Japan) and the results were normalized with *β-actin*. Amplification was performed using a reaction cycle at 95°C for 30 s, 95°C for 5 s, and 60°C for 30 s. The fluorescence signal was detected at the end of the cycle using LightCycler^®^ 480 II (Roche, Basel, Switzerland). Following amplification, melting curve or dissociation curve analysis was performed to measure the specificity of the PCR product. The temperature program used for the melting curve analysis was 95°C for 15 s followed by 60°C for 1 m and then 95 for 15 s with a ramp rate of +0.3°C/s. The relative expression was calculated using the 2^−ΔΔCt^ method, where Ct is the threshold cycle.

### Small RNA sequencing

5–40 nucleotides of small RNA fraction were collected and gel-eluted from 3% agarose gel electrophoresis. Using Agilent 2,100 Bioanalyzer, the integrity and concentration of collected small RNAs were tested and calculated. Small RNA sequencing libraries were prepared according to the manufacturer’s protocol (BGISEQ-500 Small RNA UMI Library Construction Protocol). All the samples were sequenced on BGISEQ-500 Sequencer with 50-cycle High Output Kit. Single-end reads of the 8 independent samples were trimmed for both PCR and sequencing adapters with Cutadapt. Trimmed reads were aligned to the rn6 rat reference genome using STAR (version 2.7.0e) (Dobin et al., 2013), and gene-level read counts were generated using featureCounts function from the Subread package (version 1.6.4) (Liao et al., 2014). Differentially expressed miRNAs were discovered using the edgeR (Robinson et al., 2010) package run in the R programming environment. miRNAs were considered to be significantly differentially expressed between two groups only if the false discovery rate (FDR) *p*-value was <0.05 and the absolute log2 of fold change (FC) was >1.5. The heatmap of miRNA differential expression was generated using the R language.

### RNA sequencing

The isolated total RNA was processed for preparation of an mRNA sequencing library using a TruSeq RNA Sample Preparation Kit v2 (Illumina, San Diego, CA, USA) according to the manufacturer’s instructions. In brief, mRNAs were isolated from 1 μg of total RNA on RNA purification beads by poly A capture, which was followed by enzymatic shearing. After first- and second-strand cDNA synthesis, A-tailing and end repair were performed for the ligation of proprietary primers that incorporate unique sequencing adaptors with an index for tracking Illumina reads from multiplexed samples run on a single sequencing lane. Samples were sequenced on the Illumina HiSeq 2000 platform, with paired-end 101 bp reads for mRNA sequencing using the TruSeq SBS Kit v3 (Illumina, San Diego, CA, USA). The raw imaging data were transformed by base-calling into sequence data and stored in FASTQ format. Paired-end reads of the 8 independent samples were trimmed for both PCR and sequencing adapters with Cutadapt. Trimmed reads were aligned to the rn6 rat reference genome using STAR (version 2.7.0e) (Dobin et al., 2013), and gene-level read counts were generated using featureCounts function from the Subread package (version 1.6.4) (Liao et al., 2014).

### Target gene prediction

For the computational prediction of miRNA target genes, three databases were used: TargetScan7.2 (http://www.targetscan.org/vert_72/, total context++ score < -0.2), miRanda (http://www.microrna.org/, mirSVR score < -0.1), and DIANA (http://www.microrna.gr/microT-CDS), A miTG score > 0.8.

### Plasma corticosterone

Blood samples from each sacrificed rat were collected in ethylenediaminetetraacetic acid tubes. The samples were then centrifuged at 4,000 × g at 4°C for 20 min to obtain the plasma, and plasma was collected in Eppendorf tubes and stored at -80°C. Plasma corticosterone levels were analyzed using an ELISA kit, according to the manufacturer’s protocol (Abcam, Cambridge, UK). The absorbance of the standards and samples was measured using a filter-based multi-mode microplate reader (FLUOstar Omega, BMG Labtech) at 450 nm.

### Cell culture and transfection

The human embryonic kidney cell line HEK293 was obtained from the American Type Culture Collection (ATCC, Manassas, VA, USA). Cells were cultured in Dulbecco’s modified Eagle’s medium (high glucose) supplemented with 10% fetal bovine serum (FBS, Atlas Biologicals, Fort Collins, CO, USA) and antibiotics (100 U/ml penicillin and 100 mg/ml streptomycin; HyClone Laboratories) in a humidified atmosphere containing 5% CO_2_ at 37°C. For transfection with miR-183-5p mimic, inhibitor, and negative control, cells were transfected with the above nucleotides using Oligofectamine™ reagent (Invitrogen, Carlsbad, CA, USA) according to the manufacturer’s guidelines. After 6 h of transfection, the cell culture medium was replaced with fresh medium and the cells were harvested at 48 h for the following experiments.

### Western blots

Western blot analysis was performed as described previously (Park et al., 2021). Total cellular extracts were obtained using radioimmunoprecipitation assay lysis buffer (RIPA) containing Halt Protease Inhibitor Cocktail (Thermo Fisher Scientific, Waltham, MA, USA). Next, 30 μg of total protein was loaded onto 8–13% sodium dodecyl sulfate-polyacrylamide gel (SDS-PAGE), separated by electrophoresis, and transferred onto PVDF membranes (Millipore Corp., Bedford, MA, USA). Membranes were incubated with 5% skimmed milk in TBS-T for 1 h. Membranes were then incubated overnight at 4°C with primary antibodies. After incubation with species-specific horseradish peroxidase-conjugated secondary antibodies, the proteins were visualized using SuperSignal^®^ West Dura Extended Duration Substrate (Thermo Fisher Scientific) and developed on the LAS-3000 (Fuji, Tokyo, Japan) platform according to the manufacturer’s instructions.

### Immunocytochemistry

Cells were plated onto submerged glass coverslips and seeded in 6-well plates for 24 h. After drug treatment, cells were fixed with methanol for 2 min and permeabilized with 0.1% Triton X-100 in DPBS (Dulbecco’s phosphate-buffered saline) for 10 min at room temperature (RT). Coverslips were blocked with 1% bovine serum albumin (BSA) in DPBS with Tween 20 (DPBS-T), and cells were incubated with antibodies against GR overnight at 4°C. After washing with DPBS-T and incubation with Alexa 488-conjugated secondary antibodies for 90 min at RT in the dark, nuclei were counterstained with 0.2 μg/ml DAPI. Images were obtained using a Carl Zeiss LSM 5 exciter confocal microscope (Carl Zeiss AG).

### Immunohistochemistry

Immunohistochemistry was performed as described previously (Katila et al., 2022). Rats were anesthetized by the inhalation of 2–3% isoflurane in oxygen. Under general anesthesia, the rats received intracardiac perfusion with 400 ml saline. Brains were fixed in 4% formaldehyde for examination. Tissue sections (30 μm thick) were used for immunohistochemistry. The sections were stored in an antifreeze solution before use. All selected sections were washed in KPBS (0.05 M, pH 7.4). Endogenous peroxidase activity was quenched by incubation in 3% hydrogen peroxide in methanol for 20 min and then washed five times for 10 min in KPBS. Immunohistochemical staining was performed using the avidin-biotin-peroxidase method. The sections were incubated overnight at RT with the appropriate antibodies, TH (1:500 dilution) and p-TH (1:1,000). After washing in PBS, the sections were incubated with biotinylated secondary antibodies (1:1,000; Vector Laboratories, Burlingame, CA, USA) for 90 min at RT. The sections were subsequently washed and incubated with avidin-conjugated peroxidase complex (ABC kit, 1:200; Vector Laboratories) for 30 min followed by PBS washes. The peroxidase reaction was performed in PBS using 3,3-diaminobenzidine tetrahydrochloride (DAB, 0.02%) as the chromogen. Finally, the sections were dehydrated in ethanol, cleared in xylene, mounted with Permount (Thermo Fisher Scientific), and evaluated using a light microscope (Leica, Wetzlar, Germany).

### Statistical analysis

Data were analyzed using the Student’s *t*-test, one-way analysis of variance (ANOVA) followed by Tukey’s multiple test and two-way ANOVA followed by Bonferroni’s multiple test using GraphPad Prism 8 (GraphPad Software, San Diego, CA, USA). All data are presented as mean ± standard deviation (SD) and standard error of the mean (SEM). Statistical significance was set as *p* < 0.05.

## Results

### Identification of miRNA transcripts in METH self-administered rats

**FIGURE 1:**
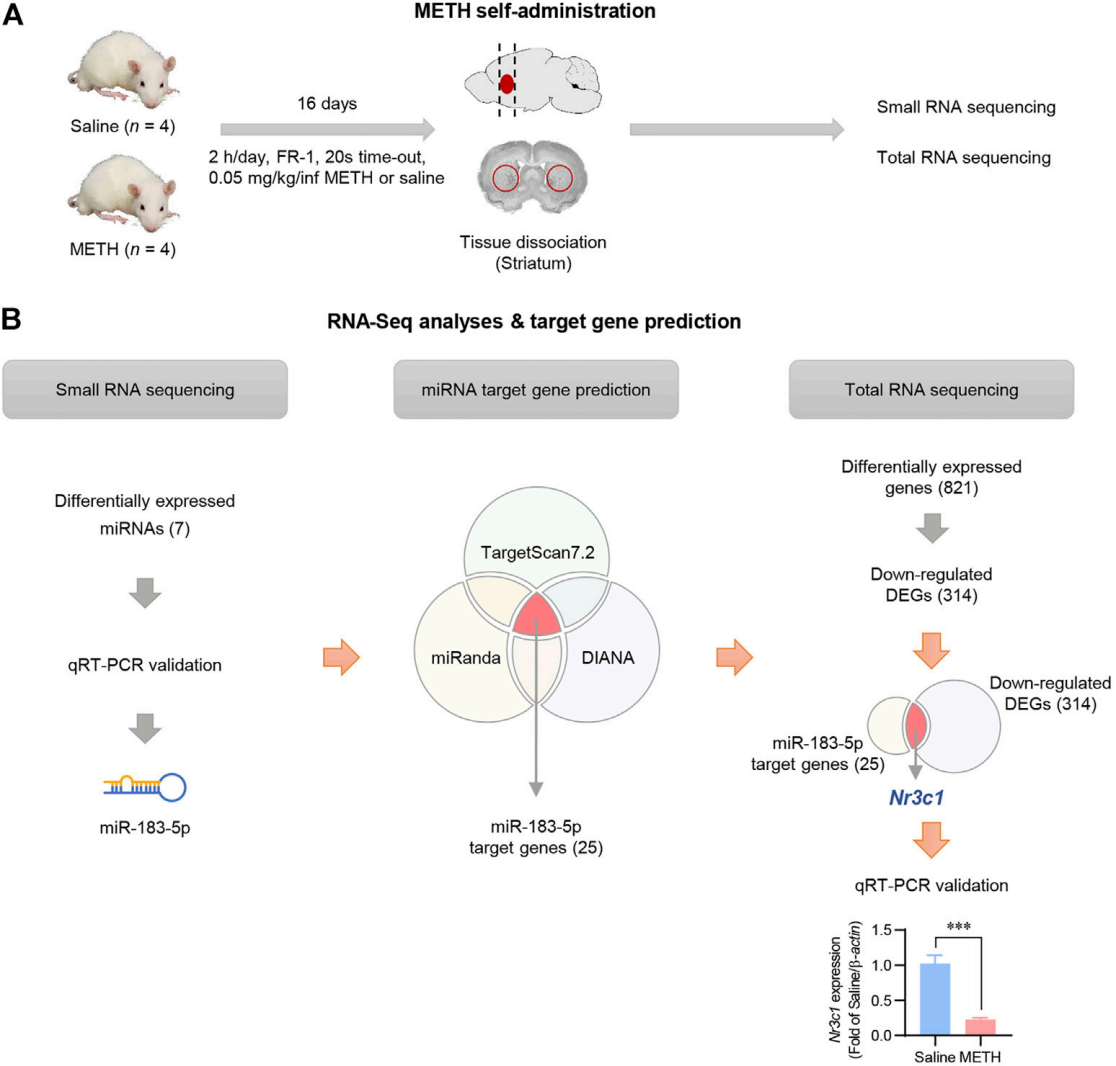
Identification of miRNA transcripts in METH self-administered rats. **(A)** Flowchart showing an overview of the experimental procedure. **(B)** The overall workflow for identification of miRNA and its target gene. *Nr3c1* expression level in the striatum was analyzed by qRT-PCR and normalized to *β-actin* level. Data are presented as the fold change relative to the saline group. Statistical analyses were performed using the Student’s *t*-test. Error bars represent mean ± SEM (*n* = 4). ^***^ *p* < 0.001.

Self-administration of METH was performed according to the experimental schedule indicated in Figure 1A. As was also demonstrated in our previous publication (Kim S. et al., 2019), two-way ANOVA with repeated measures showed a significant difference in the number of infusions between saline- and METH-administered rats. The number of infusions by METH self-administrated rats was significantly higher than that of saline self-administrated rats [F (1,96) = 261.3, *p* < 0.001] and showed <20% variation during the last 3 days of the experiment, indicating that the self-administration experiment was performed correctly (Supplementary Figure S1). After saline or METH self-administration, RNAs were isolated from the striatum and small and total RNA sequencing was performed (Figure 1A). Small RNA sequencing results showed a significant increase in seven miRNAs (False Discovery Rate [FDR] < 0.05, |fold change (FC)| ≥ 1.5, logCPM >1) in the striatum of the METH group compared to the saline group (Supplementary Table S3). The qRT-PCR analysis revealed that among the seven miRNAs, miR-183-5p and miR-182 were significantly upregulated in the striatum of METH-administered rats. Considering the higher expression levels in both small RNA sequencing and qRT-PCR analysis, we finally selected miR-183-5p and performed a follow-up experiment (Supplementary Figure S2).

Potential mRNA targets of miR-183-5p were predicted and integrated using three miRNA target prediction databases (TargetScan7.2, miRanda, and DIANA). Using an *in silico* approach, 25 candidate genes were identified, and a functional enrichment analysis was conducted (Supplementary Figure S3). Total RNA sequencing data also revealed 821 differentially expressed genes (DEGs), of which 314 DEGs were downregulated in the METH-administered rat striatum (Supplementary Table S4). To select the final target gene of miR-183-5p, we compared 314 downregulated DEGs extracted from actual experimental data with 25 target genes obtained using *in silico* prediction. *Nr3c1* was selected as the miR-183-5p target gene (Figure 1B), which was validated by qRT-PCR using three kinds of reference genes (*RNU48, 5S, and U6*) (Supplementary Figure S4).

### Effects of METH injection on locomotor activity in rats

**FIGURE 2:**
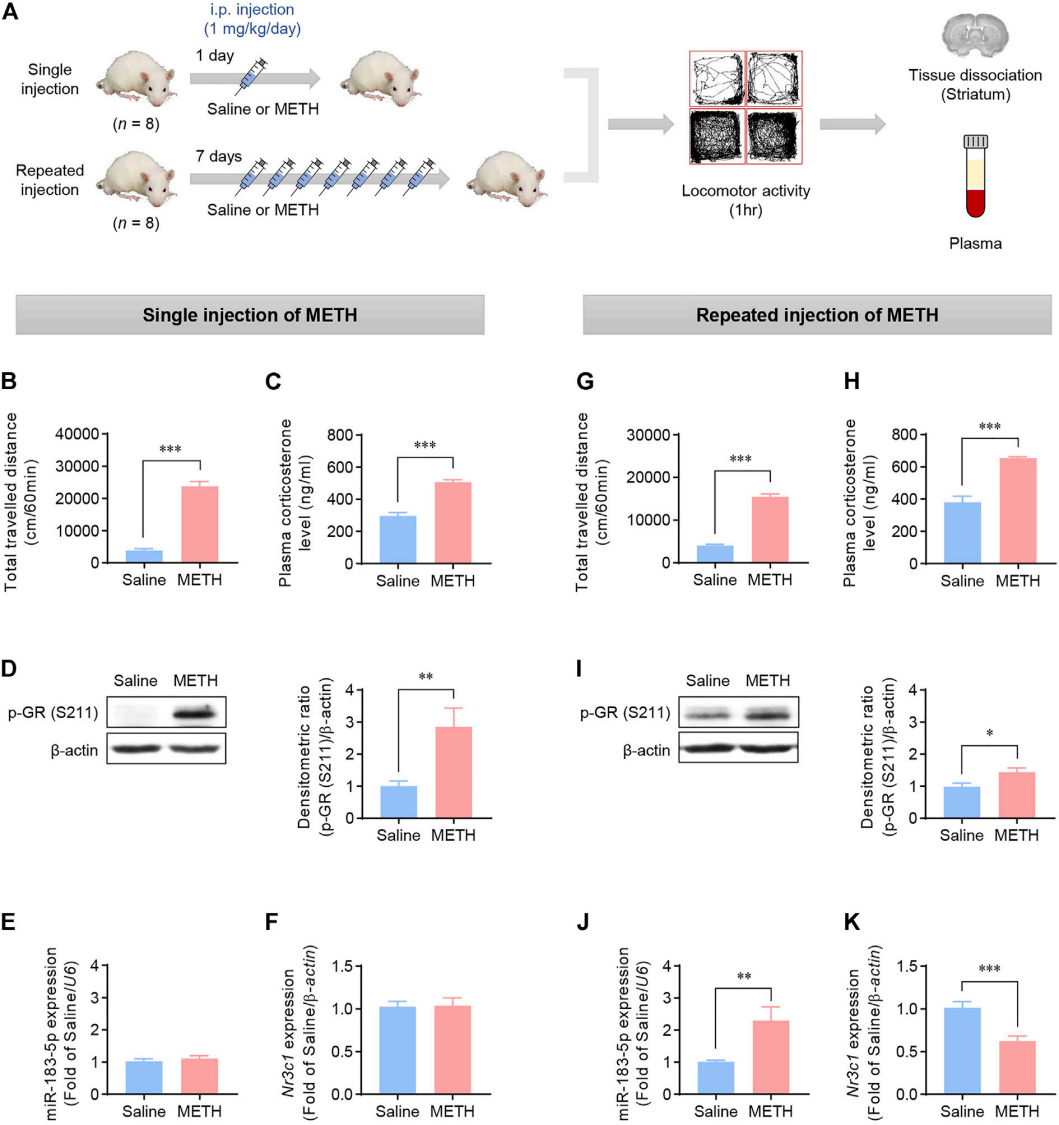
Effects of METH injections on the locomotor activity in rats. **(A)** Flowchart showing an overview of the experimental procedure. Rats were injected with single or repeated METH injections. **(B,G)** The total travelled distance was recorded 1 h after single or repeated METH injections. Statistical analyses were performed using the Student’s *t*-test. Error bars represent mean ± SEM (*n* = 8). ^***^ *p* < 0.001. **(C,H)** Plasma corticosterone levels in rats following single or repeated injections of saline or METH. Statistical analyses were performed using the Student’s *t*-test. Error bars represent mean ± SEM (*n* = 8). ^***^ *p* < 0.001. **(D,I)** Western blot analysis showing p-GR (S211) expression in rat striatum. The bands were normalized to β-actin. Quantification of the relative blot intensity of proteins was performed using ImageJ software. Data are presented as the fold change relative to the saline group. Statistical analyses were performed using the Student’s *t*-test. Error bars represent mean ± SEM (*n* = 8). ^*^ *p* < 0.05, ^**^ *p* < 0.01. **(E,J)** qRT-PCR analysis of miR-183-5p expression. The value was normalized to the *U6* level. Data are presented as the fold change relative to the saline group. Statistical analyses were performed using the Student’s *t*-test. Error bars represent mean ± SEM (*n* = 8). ^**^ *p* < 0.01. **(F,K)** qRT-PCR analysis of *Nr3c1*. The level was normalized to the *β-actin* level. Data are presented as the fold change relative to the saline group. Statistical analyses were performed using the Student’s *t*-test. Error bars represent mean ± SEM (*n* = 8). ^***^ *p* < 0.001.

We examined whether the increased miR-183-5p expression in the METH self-administration data was also present in METH-induced locomotion by investigating the effects of single and repeated METH injections on locomotion. Locomotor activity was measured after single and repeated (daily for seven consecutive days) METH injections (Figure 2A). After measuring locomotor activity, the striatum was immediately extracted to evaluate the expression of the target transcripts. The data revealed that single and repeated METH injections significantly increased locomotor activity compared with the saline group (Figures 2B,G). Additionally, a significant increase in plasma corticosterone levels (Figures 2C,H) and subsequent phosphorylation of GR (Figures 2D,I) was observed in the METH single- and repeated-injection groups. Interestingly, the single METH injection did not affect the expression of miR-183-5p or *Nr3c1* (Figures 2E,F), whereas repeated METH injections significantly increased the locomotor activity, upregulated miR-183-5p expression, and downregulated *Nr3c1* (Figures 2J,K). These results imply that single and repeated METH administrations have different effects on the expression of miR-183-5p and that repeated METH injections increase miR-183-5p expression, resulting in a decrease in GR expression, suggesting that it may play an important role in regulating the vulnerability to METH. Accordingly, we reasoned that the increased miR-183-5p might be independent of the contingency of the drug administration because it has been detected in both self-administration and repeated non-contingent METH injection.

### GC-GR signaling regulates miR-183-5p expression in HEK293 cells

**FIGURE 3:**
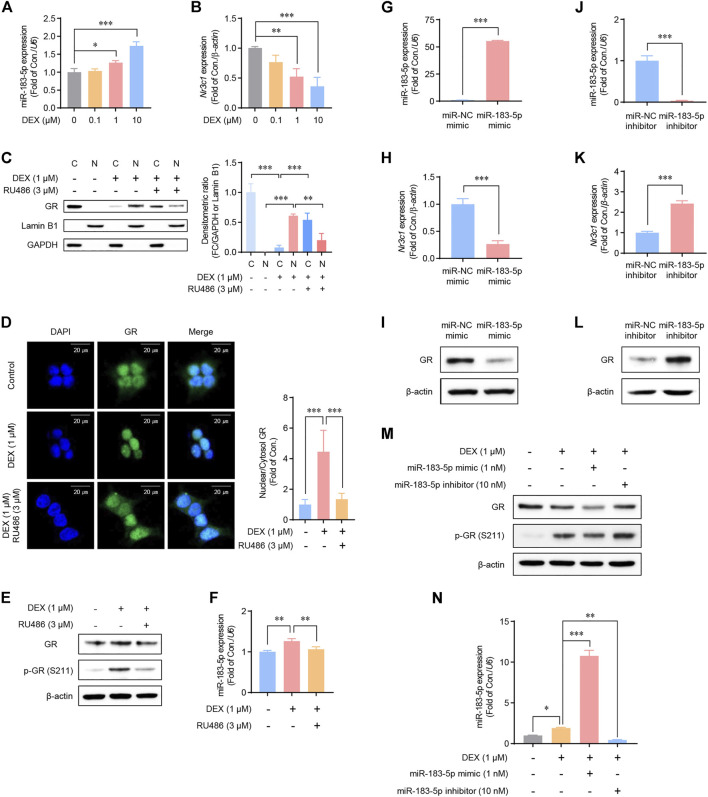
Regulation of glucocorticoid receptor (GR) and miR-183-5p expression in HEK293 cells. **(A,B)** Cells were treated with DEX as indicated. MiR-183-5p and *Nr3c1* expression level was analyzed and normalized to *U6* and *β-actin* level. Data are presented as the fold change relative to the control group. Statistical analyses were performed using the one-way ANOVA. Error bars represent mean ± SD (*n* = 3). ^*^ *p* < 0.05, ^***^ *p* < 0.001. **(C)** Western blots showing the nuclear translocation of GR protein following DEX treatment. GAPDH and Lamin B1 were used as the cytoplasmic and nuclear loading controls, respectively. Quantification of the relative blot intensity of the GR protein was performed using ImageJ software. Statistical analyses were performed using the one-way ANOVA. Error bars represent mean ± SD (*n* = 3). ^**^ *p* < 0.01, ^***^ *p* < 0.001. **(D)** Cells seeded on sterile coverslips were treated with DEX for 6 h, then fixed with methanol, incubated with fluorescently tagged mouse monoclonal GR antibody, and stained with Alexa Flour 488 (green)-labeled anti-mouse antisera and DAPI. GR signals were then visualized by confocal microscopy. Quantification of the relative fluorescence intensity of GR was performed using ImageJ software. Data are presented as the fold change relative to the control group. Statistical analyses were performed using the one-way ANOVA. Error bars represent mean ± SD (*n* = 3–6). ^**^ *p* < 0.01. **(E)** Cells were treated with 1 μM of DEX and 3 μM of RU486 as indicated for 24 h. **(F)** Cells were treated with DEX and RU486 as indicated. MiR-183-5p expression level was analyzed and normalized to *U6* level. Data are presented as the fold change relative to the control group. Statistical analyses were performed using the one-way ANOVA. Error bars represent mean ± SD (*n* = 3). ^**^ *p* < 0.01. **(G,H)** Cells were treated with 1 nM miR-183-5p mimic for 48 h. MiR-183-5p and *Nr3c1* expression level was analyzed by qRT-PCR and normalized to *U6* and *β-actin* level. Data are presented as the fold change relative to the control group. Statistical analyses were performed using the Student’s *t*-test. Error bars represent mean ± SD (*n* = 3). ^***^ *p* < 0.001. **(I)** GR protein expression level. **(J,K)** Cells were treated with 10 nM miR-183-5p inhibitor for 48 h. MiR-183-5p and *Nr3c1* expression level was analyzed by qRT-PCR and normalized to *U6* and *β-actin* level. Data are presented as the fold change relative to the control group. Statistical analyses were performed using the one-way ANOVA. Error bars represent mean ± SD (*n* = 3). ^***^ *p* < 0.001. **(I,L)** GR protein expression level. **(M)** Cells were treated with the indicated combinations of DEX, miR-183-5p mimic, and miR-183-5p inhibitor, and GR protein expressions were measured by western blot analyses. **(N)** miR-183-5p expression level was analyzed and normalized to *U6* level. Data are presented as the fold change relative to the control group. Statistical analyses were performed using the one-way ANOVA. Error bars represent mean ± SD (*n* = 3). ^*^ *p* < 0.05, ^**^ *p* < 0.01, ^***^ *p* < 0.001.

Our data revealed that METH injections increase the plasma corticosterone level, and the expression of miR-183-5p and GR appeared to be inversely regulated in the repeated-METH-injection group. Therefore, we hypothesized that a negative feedback loop could exist between miR-183-5p and GR and that miR-183-5p expression would be regulated via GC-GR signaling. GR is phosphorylated through ligand binding and acts as a TF *via* nuclear translocation (Scheschowitsch et al., 2017). Therefore, we performed experiments to confirm the role of GC-GR signaling in miR-183-5p expression following treatment with dexamethasone (DEX), a well-known agonist of GR, in HEK293 cell lines. DEX treatment resulted in a dose-dependent increase in miR-183-5p expression (Figure 3A) and a decrease in GR gene expression (Figure 3B). We then examined the nuclear translocation of GR following co-treatment with DEX and RU486, an antagonist of GR. DEX induced the nuclear translocation of GR, whereas co-treatment with RU486 attenuated it (Figure 3C). This was further confirmed by immunofluorescence analysis (Figure 3D). Moreover, the DEX-induced increases in phosphorylated-GR (Figure 3E) and miR-183-5p levels (Figure 3F) were significantly decreased by RU486 treatment. These results demonstrate that miR-183-5p expression is regulated by GC-GR signaling.

### MiR-183-5p downregulates GR expression in HEK293 cells

To determine whether miR-183-5p regulates GR mRNA and protein expression, HEK293 cells were transfected with either a miR-183-5p mimic or inhibitor. Our data revealed that the GR mRNA and protein expression levels were decreased by the miR-183-5p mimic (Figures 3G–I) but increased by the miR-183-5p inhibitor (Figures 3J–L). Interestingly, DEX induced GR phosphorylation but decreased total protein expression, which was further decreased by the miR-183-5p mimic but not by the inhibitor (Figure 3M). In these conditions, the expression of miR-183-5p by mimic and inhibitor treatment was verified in Figure 3N. Therefore, these results suggest that a negative feedback loop may exist between miR-183-5p and GR, suggesting that the transcriptionally increased miR-183-5p expression, by GC-GR signaling, inhibits GR expression.

### Effects of striatal miR-183-5p on METH-induced locomotion

**FIGURE 4:**
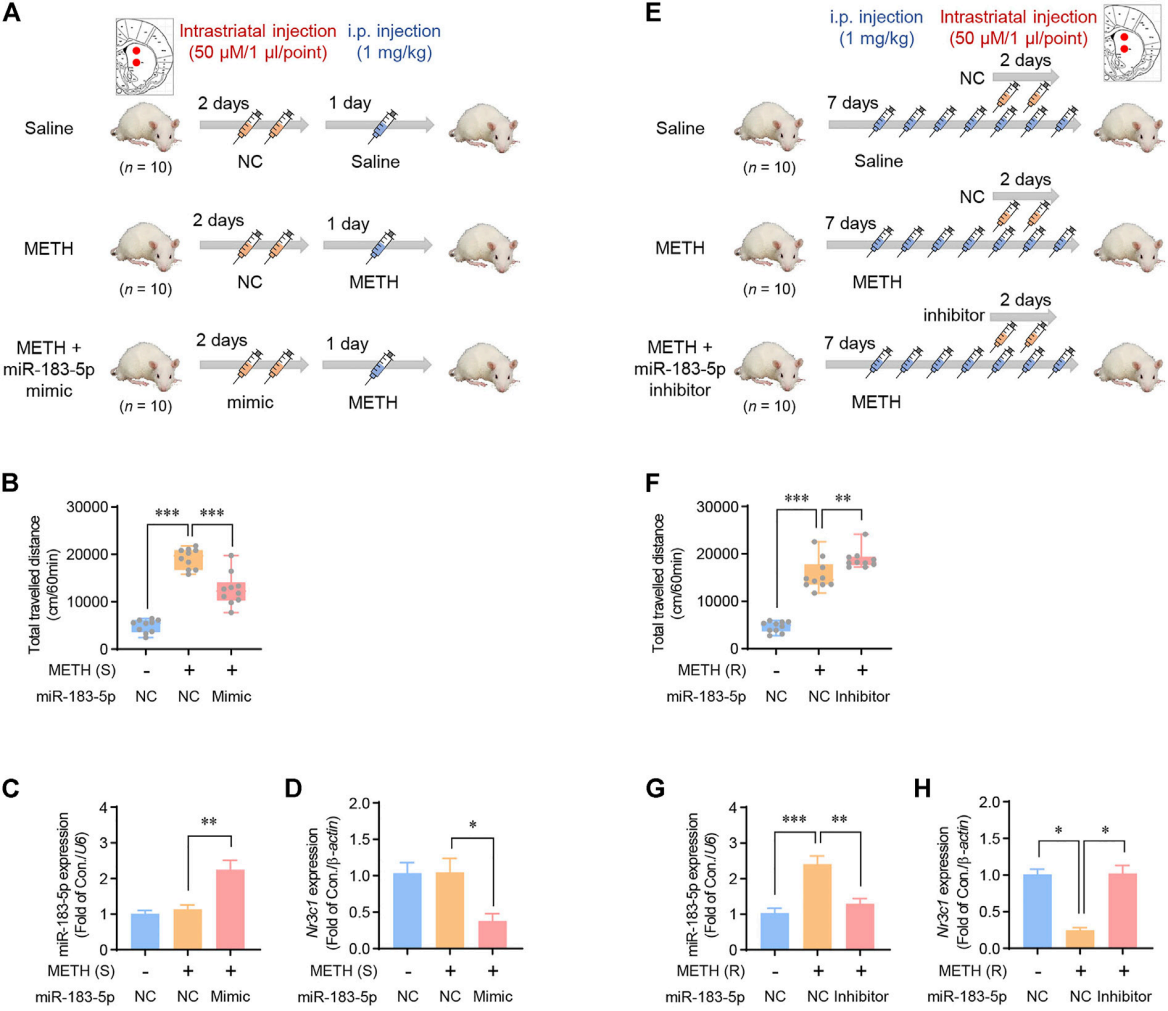
Effects of striatal miR-183-5p on METH-induced locomotor activity. **(A)** Flowchart showing an overview of a single METH injection. Rats were injected with vehicle or miR-183-5p mimic twice before METH injection. **(B)** The locomotor activities of rats were recorded 1 h after METH injection. Statistical analysis was performed using the one-way ANOVA. Error bars represent mean ± SEM (*n* = 10). ^**^ *p* < 0.01, ^***^ *p* < 0.001. **(C,D)** MiR-183-5p and *Nr3c1* expression level in the striatum was analyzed and normalized to *U6* and *β-actin* level. Data are presented as the fold change relative to the control group. Statistical analyses were performed using the one-way ANOVA. Error bars represent mean ± SEM (*n* = 5). ^*^ *p* < 0.05, ^**^ *p* < 0.01. **(E)** Flowchart showing an overview of repeated METH injections. Rats were injected with vehicle or miR-183-5p inhibitor on the 5th and 6th days following METH injections. **(F)** The locomotor activities of rats were recorded 1 h after METH injection. Statistical analyses were performed using the one-way ANOVA. Error bars represent mean ± SEM (*n* = 5). ^**^ *p* < 0.01, ^***^ *p* < 0.001. **(G,H)** MiR-183-5p and *Nr3c1* expression level in the striatum was analyzed and normalized to *U6* and *β-actin* level. Data are presented as the fold change relative to the control group. Statistical analyses were performed using the one-way ANOVA. Error bars represent mean ± SEM (*n* = 5). ^*^ *p* < 0.05, ^**^ *p* < 0.01, ^***^ *p* < 0.001.

Based on the data on miR-183-5p expression according to the duration of METH administration (Figure 2), the miR-183-5p mimic and inhibitor were stereotaxically injected at the designated location in the striatum to determine the role of miR-183-5p in METH-induced locomotion. The mimic was co-injected into the single-METH-injection group, in which miR-183-5p expression was not changed, and the inhibitor was co-injected in the repeated-METH-injections group, in which miR-183-5p expression was increased. The procedure for the single-injection model is shown in Figure 4A. In the single-METH-injection group, the enhanced METH-induced locomotor activity was significantly attenuated by co-injection with the mimic (Figure 4B). Consistent with the previous results, a single METH injection alone did not change the expression of miR-183-5p and GR, whereas the co-injection of METH with the miR-183-5p mimic significantly decreased GR expression (Figures 4C,D). To investigate the effect of miR-183-5p suppression in the striatum on METH-induced locomotor activity, we performed experiments using a repeated-injection rat model (Figure 4E). In the repeated-METH-injection group, the increased METH-induced locomotor activity was further enhanced by the miR-183-5p inhibitor (Figure 4F). Repeated METH injections significantly increased miR-183-5p expression and decreased GR expression; however, these changes were reversed by striatal co-injection with the miR-183-5p inhibitor (Figures 4G,H). Therefore, these results suggest a possible role of striatal miR-183-5p in METH-induced locomotion.

### MiR-183-5p attenuates TH phosphorylation in the striatum

**FIGURE 5:**
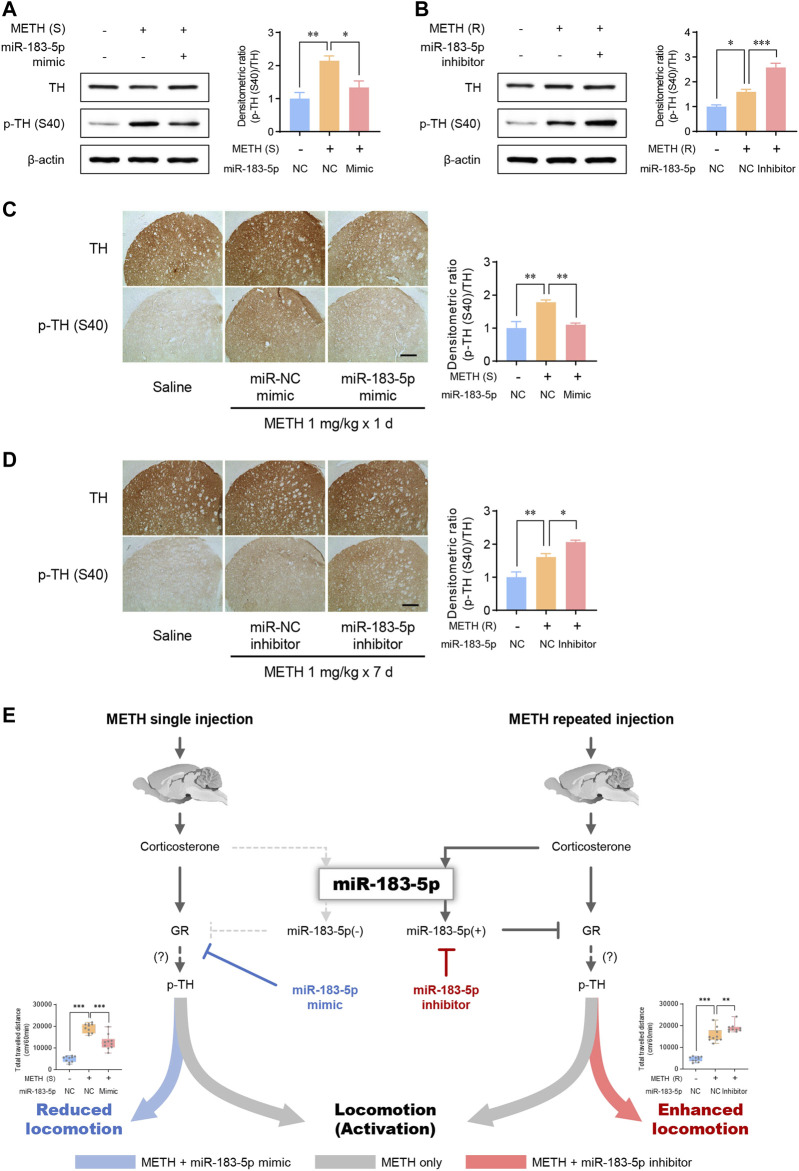
MiR-183-5p downregulated GR-TH signaling in the striatum of rats. **(A,B)** Western blot analysis of TH and p-TH (S40) expression in the striatum. The immunoblots were normalized to β-actin. Quantification of the relative blot intensity of proteins was performed using ImageJ software. Data are presented as the fold change relative to the control group. Statistical analyses were performed using the one-way ANOVA. Error bars represent mean ± SEM (*n* = 5). ^*^ *p* < 0.05, ^**^ *p* < 0.01. **(C,D)** Immunohistochemical detection of TH and p-TH (S40) in the striatum. Scale bar = 500 μm. Quantification of the relative intensity of proteins was performed using ImageJ software. Data are presented as the fold change relative to the control group. Statistical analyses were performed using the one-way ANOVA. Error bars represent mean ± SEM (*n* = 5). ^*^ *p* < 0.05, ^**^ *p* < 0.01. **(E)** Proposed mechanism by which miR-183-5p controls METH-induced locomotor activity.

Tyrosine hydroxylase (TH), a rate-limiting enzyme in catecholamine synthesis, is activated by psychostimulants and induces rewarding effects by promoting DA synthesis (Yao et al., 2010; Botanas et al., 2017). We performed western blots and immunohistochemistry to determine whether TH phosphorylation (p-TH) is involved in METH-induced locomotion. Our data revealed that both single and repeated injections of METH significantly increased p-TH, indicating the involvement of TH in enhanced METH-induced locomotor activity (Figures 5A,B). The miR-183-5p mimic decreased p-TH levels in the striatum in the single-METH-injection group (Figure 5A). Similarly, the p-TH level was further enhanced by miR-183-5p inhibition (Figure 5B). In the current study, immunohistochemistry analysis confirmed that the p-TH level was significantly decreased by the miR-183-5p mimic but increased by the miR-183-5p inhibitor (Figures 5C,D). Therefore, these data suggest that the increase in miR-183-5p, induced by repeated METH injections, reduces METH-induced locomotor activity by attenuating TH phosphorylation in the striatum.

## Discussion

We investigated the role of miRNAs in gene regulatory mechanisms in the striatum, which is involved in METH self-administration. The use of addictive drugs changes the expression patterns of miRNAs in the brain, which are involved in reward and behavior changes as a result of drug usage. Chronic METH use robustly changes the expression of various miRNAs and target genes in the NAc of mice (Zhu et al., 2015). In current study, we profiled novel changes in miRNAs and mRNAs in the striatum of METH-self-administered rats using RNA sequencing analysis. We revealed a significant increase in miR-183-5p expression in the striatum of METH self-administered rats. Based on predicted databases from TargetScan7.2, miRanda, and DIANA, we focused on *Nr3c1* (GR), one of the target genes of miR-183-5p. *In vivo* and cell experiments using a miR-183-5p mimic and inhibitor confirmed that GR expression was possibly regulated by miR-183-5p (Figures 3, 4). Additionally, the positive or negative regulation of miR-183-5p altered METH-induced locomotor activity (Figures 4B,F). Therefore, we suggest that miR-183-5p, which was increased by repeated METH administration, may play a role as an important regulator of METH-induced locomotion and self-administration.

Psychostimulants, such as METH, cause a stress response by activating the neuroendocrine system, including the HPA axis, leading to physiological and behavioral reactions (Manetti et al., 2014; Zuloaga et al., 2015). GCs play a role in regulating CNS activity by regulating homeostatic disturbance. Additionally, GR is involved in dopamine release (Piazza et al., 1991). Individuals with higher GCs responses to METH showed more positive subjective drug responses than those with relatively lower GCs responses (Oswald et al., 2005). In other words, GCs are involved in the individual tendencies to develop addictive behavior, which can explain individual differences in drug sensitivity (de Jong and de Kloet, 2004). This indicates that an increase in GCs through action on mesodermal dopaminergic neurons can increase the sensitivity to drug abuse. In healthy individuals, the administration of GCs increases drug-induced compensation levels and promotes amphetamine self-administration (Piazza et al., 1991). Conversely, the administration of RU486, an antagonist of GR, reduces the urge to self-administer cocaine or amphetamine in mice (Fiancette et al., 2010; Stairs et al., 2011). A decrease in GR mRNA levels in the brains of transgenic mice decreased the sensitivity to cocaine (St-Hilaire et al., 2003). In addition, selectively inactivated GR in dopaminergic neurons leads to reduced amphetamine and cocaine-seeking behaviors (Ambroggi et al., 2009; Parnaudeau et al., 2014). In our study, a miR-183-5p mimic induced GR downregulation and a reduction in METH-induced locomotor activity (Figure 4B), indicating the role of GR in behavioral changes induced by addictive drugs. Therefore, GC-GR signaling in the striatum affects the self-administration impulses of psychostimulants, including METH, and plays a pivotal role in the initial stage leading to drug addiction caused by repeated drug use.

Consistent with previous findings, we observed that plasma corticosterone levels increased after METH injection (Avila et al., 2003; Harris et al., 2003; Herring et al., 2008; Ago et al., 2009). In particular, a significantly increased corticosterone level was detected in the plasma of single and repeatedly injected METH groups. In these conditions, both single and repeated METH injections significantly increased the phosphorylation of GR and locomotor activity. Therefore, it is reasonable to infer that an increase in GCs may increase the sensitivity to METH, thereby increasing METH-induced locomotion. Thus, we were initially surprised by the contradictory effect of miR-183-5p on METH-induced locomotion, as shown in experiments with mimic and inhibitor. The injection of mimic and inhibitor of miR-183-5p significantly reduced and enhanced METH-induced locomotor activity in single and repeated METH injection models, respectively. What do these results signify? Perhaps the role of miR-183-5p, which increases on repeated METH administration, may be a kind of compensatory mechanism that reduces sensitivity to drug in METH abuse situations (Figure 5E). In future studies, it needs to be verified which mechanism increases miR-183-5p by repeated METH administration and exhibits a compensatory effect on METH-induced locomotion.

Moreover, DEX has been reported to activate GR in several cell lines (Funato et al., 2006; Blind and Garabedian, 2008; Chen et al., 2008), and this activation downregulates GR expression (Michas et al., 2011; Son et al., 2015; Lea et al., 2020). DEX-induced reduced GR expression may be due to a decrease in the half-life of GR mRNA, control of the turnover rate of GR protein, and proteasomal degradation (Dong et al., 1988; Wallace and Cidlowski, 2001). Additionally, recent reports have suggested that miR-124a and miR-142-3p decrease the translation of GR (Vreugdenhil et al., 2009; Lv et al., 2012). Importantly, decreased GR mRNA and protein expression levels were detected in the blood of cocaine users and the brains of animals administered with addictive drugs (Lowy and Novotney, 1994; Mantsch et al., 2007; Schote et al., 2019). These lines of evidence strengthen our hypothesis that METH-induced increased GC secretion can lead to a decrease in GR expression through miR183-5p. Therefore, we suggest that the METH-induced increase in miR-183-5p levels via the GC-GR signaling pathway may play a role in the pathogenesis of METH addiction as a novel regulatory mechanism. Moreover, an increase in miR-183-5p and a decrease in GR expression in the striatum are potential molecular indicators for diagnosing whether addictive drugs such as METH were used; however, this should be verified in future studies.

Our results show that miR-183-5p was increased by repeated METH injections, which reduced METH-induced locomotor activity by downregulating GR and p-TH (Figure 5). However, how TH phosphorylation can be regulated by miR183-5p remains unknown. Inhibition of TH phosphorylation by miR-183-5p can be explained through several possible mechanisms. The first possibility is that an increase in corticosterone secretion by METH may increase TH phosphorylation through GR activation. Although no direct data were presented in this study, previous studies have shown that DEX treatment increases TH mRNA levels and TH activity (Joseph et al., 1998; Makino et al., 2002; Soto-Pina et al., 2016), which suggests the possibility of TH phosphorylation through GR activation. Another possibility also may exist. The role of DA in behavioral reinforcement by psychostimulants has been reported in many previous studies (Yokel and Wise, 1975; Berridge and Robinson, 1998; Pessiglione et al., 2006). Also, studies suggest that TH activity could be regulated through DA signaling. For example, it is well known that the dopamine D_2_ receptor (DRD_2_) regulates the phosphorylation of TH at Ser40 in rat striatum (Lindgren et al., 2001). Furthermore, acute or chronic treatment with a conventional neuroleptic, haloperidol, has shown to regulate the phosphorylation of striatal TH and dopamine turnover *via* blockade of DRD_2_ (Salvatore et al., 2000; Håkansson et al., 2004). Therefore, it is suggested that miR-183-5p may modulate diverse dopaminergic synapse events, including DRD_2_ regulation, after repeated METH administration. In this regard, our functional enrichment analysis of 25 potential targets of miR-183-5p showed that the ‘AMPK signaling pathway’ and ‘dopaminergic synapse’ were identified as two first terms for KEGG, indicating the involvement of DA signaling in miR-183-5p regulated TH activity (Supplementary Figure S3).

In summary, we identified a novel miRNA, miR-183-5p, in the striatum of METH-self-administered rats through transcriptome profiling and investigated its role in METH-induced locomotion. Our results show that striatal miR-183-5p might contribute to the reduction of METH-induced locomotion through the downregulation of GR and p-TH. Collectively, these findings reveal a novel mechanism of miR-183-5p in the striatum that controls behavioral and molecular responses to METH.

## Data Availability

The total RNA (accession number GSE211184) and small RNA (accession number GSE211185) sequencing data analyzed during the current study are available in the Gene Expression Omnibus (GEO) database.
