# Supplementary material for: Striatal miR-183-5p inhibits methamphetamine-induced locomotion by regulating glucocorticoid receptor signaling
Source: Front Pharmacol. 2022 Sep 26;13:997701. doi: 10.3389/fphar.2022.997701 (PMC9549132; doi:10.3389/fphar.2022.997701)
Supplement: Supplementary file 2 [file DataSheet1.docx]

Supplementary Material

# Supplementary Figures

**
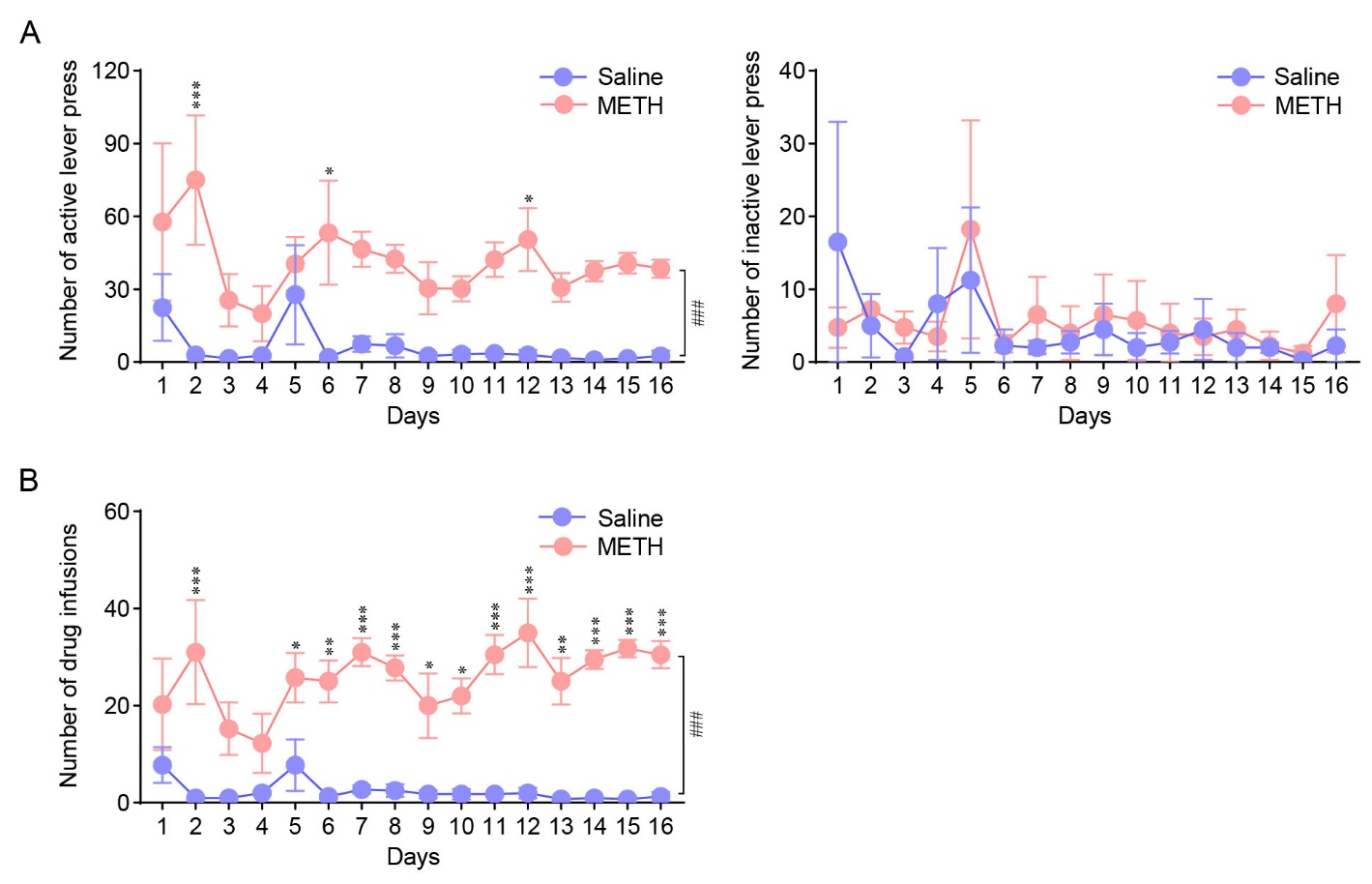
**

**Supplementary Figure 1. The number of lever press and drug infusion of saline and METH self-administered rats** (A) The numbers of active and inactive lever presses by saline or METH self-administration. (B) The number of infusions during METH self-administration at 0.05 mg/kg (2 h/day, fixed-rate 1 (FR1), 20 s time-out). Data were represented as mean ± SEM (n = 4/group) and were subjected to two-way ANOVA (F(1,96)=261.3, ^###^ *p* < 0.001), followed by Bonferroni’s multiple comparison post hoc test (^*^ *p* < 0.05, ^**^ *p* < 0.01, ^***^ *p* < 0.001. vs saline group per each day).


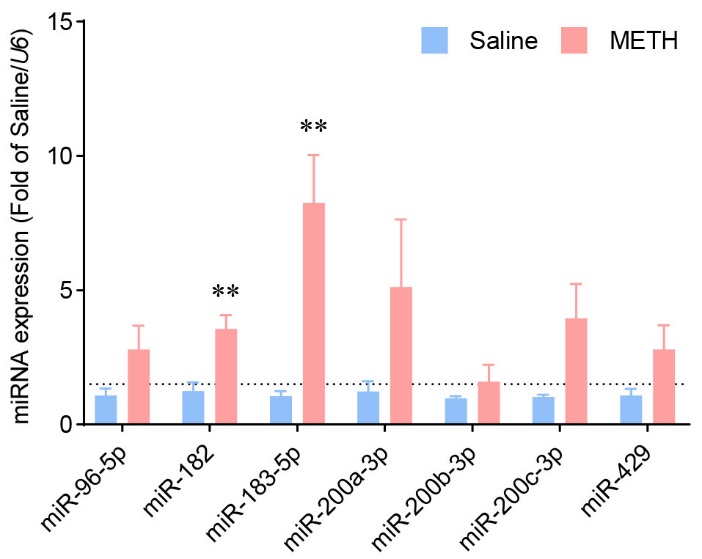


**Supplementary Figure 2. Expression of differentially expressed miRNAs in the striatum of METH self-administered rats.** Seven DEMs expression levels in the striatum were analyzed using qRT-PCR and normalized to *U6* level. Data are presented as the fold change relative to the saline group. Statistical analyses were performed using the Student’s *t*-test. Error bars represent mean ± SEM (*n* = 4). ^**^ *p* < 0.01.

**
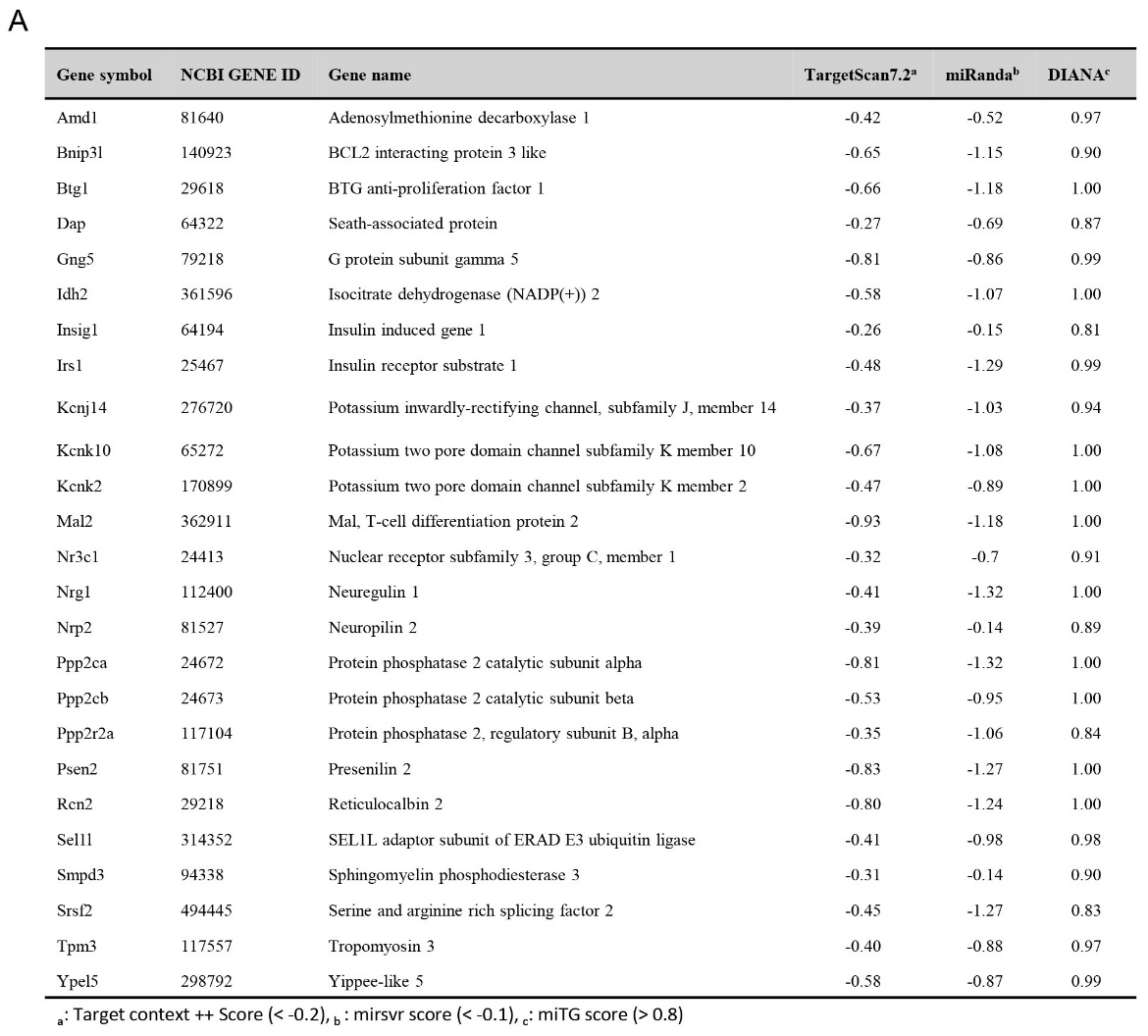
**

**
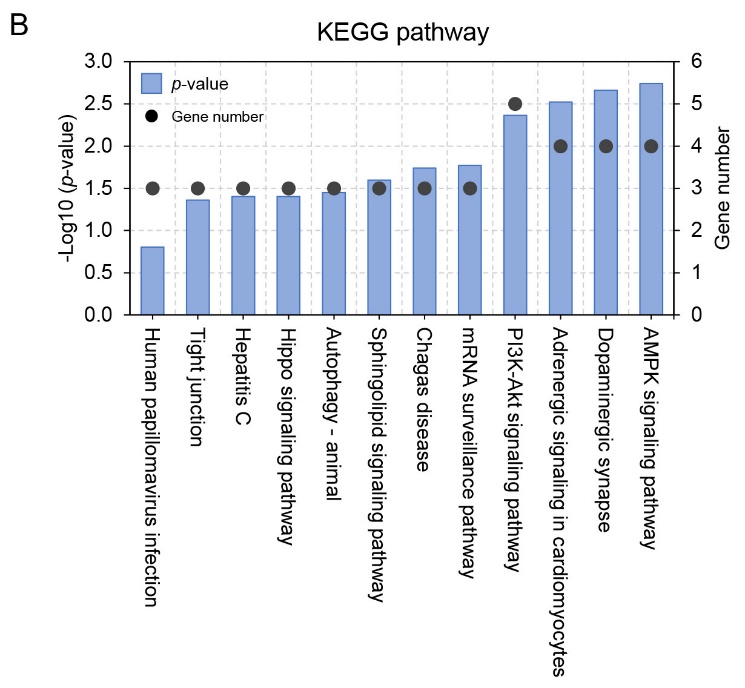
**

**Supplementary Figure 3. miR-183-5p target genes and enrichment analysis** (A) List of potential mRNA targets of miR-183-5p (B) Functional enrichment analysis of mRNA targets of miR-183-5p in striatum. Kyoto Encyclopedia of Genes and Genomes (KEGG) pathway were analyzed miR-183-5p target genes using Database for Annotation, Visualization, and Integrated Discovery (DAVID) database. The horizontal axis represents various functional terms, including KEGG pathway. The vertical axis represents the *p*-value (-Log10) and the number of genes enriched in KEGG term.

**
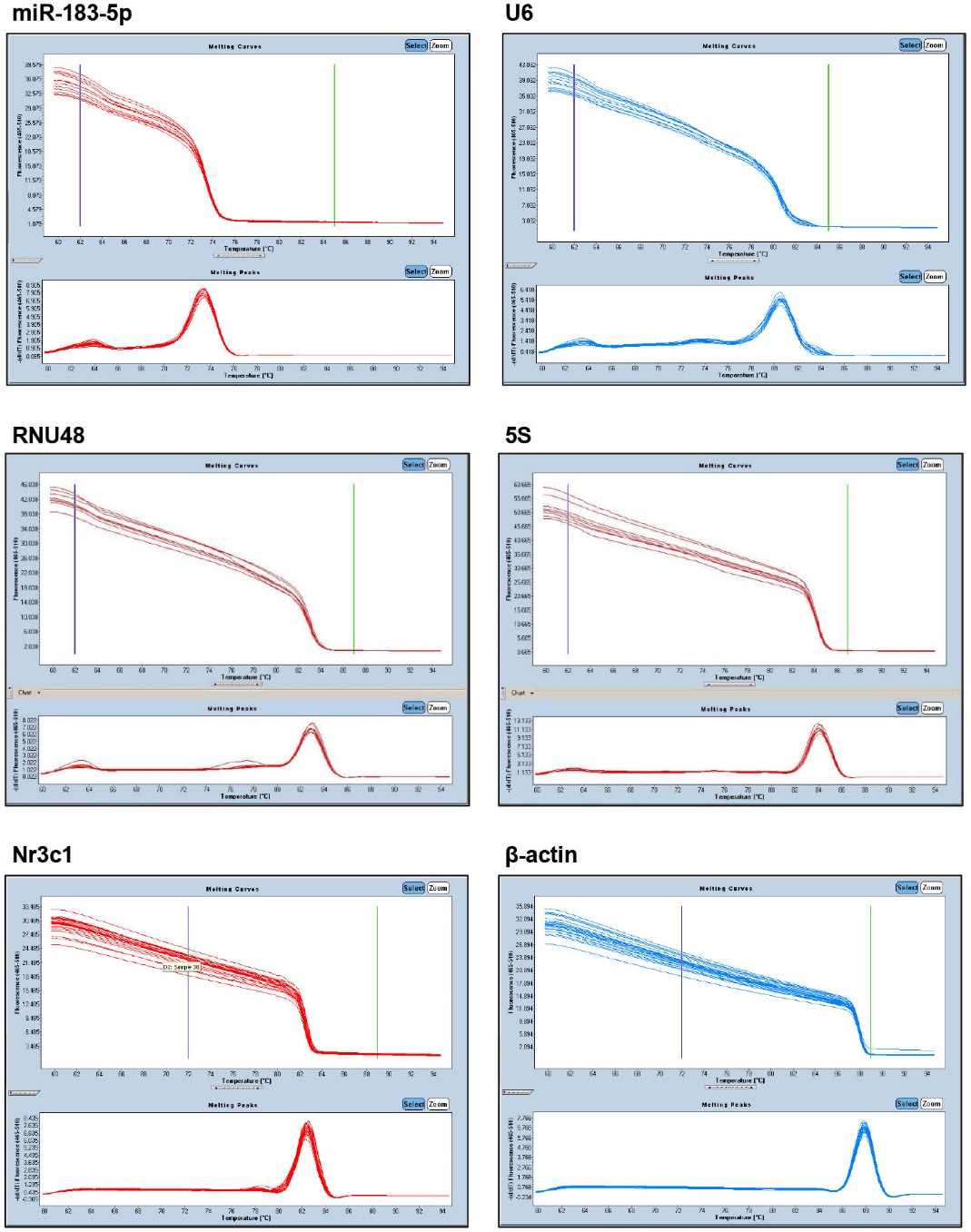
**

**Supplementary Figure 4. Melting curve analysis of PCR products.** cDNA samples were amplified in real-time PCR with specific set of primers and melting curve analysis was performed to confirm the specificity of the PCR products. Melting curve analysis of the amplified products demonstrated a single, sharp peak, which indicated good specificity.

**Supplementary Table 1. Sequence of miR-183-5p mimic, inhibitor, and negative control**

| **Names** | **Sequences** |
| --- | --- |
| miR-183-5p mimic | 5’-UAUGGCACUGGUAGAAUUCACU-3’ |
| miR-183-5p inhibitor | 5’-GTGAATTCTACCATGTGCCAT-3’ |
| Negative control (mimic) | 5'-UCACCGGGUGUAAAUCAGCUUG-3' |
| Negative control (inhibitor) | 5'-TAACACGTCTATACGCCCA-3' |

**Supplementary Table 2. Sequence of primers**

| **Genes** | **Primer Sequences** |
| --- | --- |
| miR-183-5p | 5'-UAUGGCACUGGUAGAAUUCACU-3' |
| *U6* (F) | 5'-TGCTTCGGCAGCACATGTGC-3' |
| *U6* (R) | 5'-AGGGGCCATGCTAATCTTCT-3' |
| *RNU48* (F) | 5'-TCTGAGTGTCTTCGCTGACG-3' |
| *RNU48* (R) | 5'-GAGGTATTCGCACCAGAGGA-3' |
| *5S* (F) | 5'-TCTCGTCTGATCTCGGAAGC-3' |
| *5S* (R) | 5'-AGCCTACAGCACCCGGTATT-3' |
| *Nr3c1* (F) | 5'-GTCATTACGGGGTGCTGACA-3' |
| *Nr3c1* (R) | 5'-GCTGGGCAGTTTTTCCTTCG-3' |
| *β-actin* (F) | 5'-TCCCTGTATGCCTCTGGTCG-3' |
| *β-actin* (R) | 5'-ATCCTGTCAGCAATGCCTGG-3' |

**Supplementary Table 3. List of differentially expressed miRNAs (7 miRNAs)**

| **Up-regulated DEMs (12 miRNAs)** | | | | |
| --- | --- | --- | --- | --- |
| **Name** | **Accession** | **LogCPM** | **Log2FC** | **FDR (adjusted p-value)** |
| rno-miR-183-5p | MIMAT0000860 | 6.1 | 4.2 | 0.000 |
| rno-miR-96-5p | MIMAT0000818 | 5.3 | 3.5 | 0.000 |
| rno-miR-182 | MIMAT0005300 | 5.8 | 3.6 | 0.000 |
| rno-miR-429 | MIMAT0001538 | 6.8 | 4.5 | 0.000 |
| rno-miR-200b-3p | MIMAT0000875 | 4.9 | 4.5 | 0.001 |
| rno-miR-200a-3p | MIMAT0000874 | 6.6 | 4.3 | 0.002 |
| rno-miR-200c-3p | MIMAT0000873 | 3.9 | 3.0 | 0.009 |
| rno-miR-200b-5p | MIMAT0017152 | 0.9 | 3.4 | 0.002 |
| rno-miR-141-3p | MIMAT0000846 | 0.4 | 2.9 | 0.042 |
| rno-miR-183-3p | MIMAT0017141 | 0.1 | 3.4 | 0.002 |
| rno-miR-200a-5p | MIMAT0017151 | 0.0 | 3.4 | 0.025 |
| rno-miR-141-5p | MIMAT0017128 | -0.4 | 4.3 | 0.003 |

**Supplementary Table 4. List of differentially expressed genes (821 genes)**

| **Up-regulated DEGs (507 genes)** | | | | | |
| --- | --- | --- | --- | --- | --- |
| **Symbol** | **EntrezID** | **Description** | **Log2FC** | **logCPM** | **FDR (adjusted *p*-value)** |
| Fcrl2 | 310694 | Fc receptor-like 2 | 3.7 | 1.4 | 0.026 |
| Oxt | 25504 | oxytocin/neurophysin I prepropeptide | 3.6 | 1.9 | 0.009 |
| Mir132 | 100314029 | microRNA 132 | 2.8 | 1.5 | 0.000 |
| Nr4a3 | 58853 | nuclear receptor subfamily 4, group A, member 3 | 2.8 | 4.4 | 0.000 |
| Hspa1b | 24472 | heat shock protein family A (Hsp70) member 1B | 2.6 | 1.6 | 0.000 |
| Egr2 | 114090 | early growth response 2 | 2.5 | 4.4 | 0.002 |
| Mir212 | 100314247 | microRNA 212 | 2.1 | 1.1 | 0.000 |
| Fosl2 | 25446 | FOS like 2, AP-1 transcription factor subunit | 2.0 | 3.0 | 0.000 |
| Nr4a1 | 79240 | nuclear receptor subfamily 4, group A, member 1 | 1.9 | 7.1 | 0.002 |
| Hspa1a | 294254 | heat shock protein family A (Hsp70) member 1A | 1.9 | 1.8 | 0.000 |
| Arc | 54323 | activity-regulated cytoskeleton-associated protein | 1.8 | 7.8 | 0.003 |
| Fos | 314322 | Fos proto-oncogene, AP-1 transcription factor subunit | 1.7 | 4.0 | 0.009 |
| Egr4 | 25129 | early growth response 4 | 1.7 | 7.6 | 0.000 |
| Dusp5 | 171109 | dual specificity phosphatase 5 | 1.5 | 3.3 | 0.000 |
| Junb | 24517 | JunB proto-oncogene, AP-1 transcription factor subunit | 1.5 | 8.0 | 0.000 |
| Gpr3 | 266769 | G protein-coupled receptor 3 | 1.5 | 2.0 | 0.002 |
| Pkp2 | 287925 | plakophilin 2 | 1.4 | 2.1 | 0.000 |
| Dusp4 | 60587 | dual specificity phosphatase 4 | 1.4 | 2.6 | 0.001 |
| Fosb | 100360880 | FosB proto-oncogene, AP-1 transcription factor subunit | 1.3 | 4.6 | 0.000 |
| Ctrl | 117184 | chymotrypsin-like | 1.1 | 1.4 | 0.032 |
| Plk3 | 58936 | polo-like kinase 3 | 1.1 | 2.9 | 0.012 |
| Trib1 | 78969 | tribbles pseudokinase 1 | 1.1 | 3.3 | 0.008 |
| RGD1564664 | 499839 | similar to LOC387763 protein | 1.1 | 7.4 | 0.000 |
| Ier5 | 498256 | immediate early response 5 | 1.1 | 6.4 | 0.000 |
| Hspb1 | 24471 | heat shock protein family B (small) member 1 | 1.0 | 3.7 | 0.000 |
| Egr1 | 24330 | early growth response 1 | 1.0 | 7.0 | 0.003 |
| Pdlim3 | 114108 | PDZ and LIM domain 3 | 1.0 | 3.5 | 0.004 |
| Ptgs2 | 29527 | prostaglandin-endoperoxide synthase 2 | 1.0 | 2.8 | 0.003 |
| Rem2 | 64626 | RRAD and GEM like GTPase 2 | 1.0 | 6.8 | 0.000 |
| Nrn1l | 100189544 | neuritin 1-like | 1.0 | 2.8 | 0.010 |
| Pcsk1 | 25204 | proprotein convertase subtilisin/kexin type 1 | 1.0 | 3.5 | 0.000 |
| Slc25a25 | 246771 | solute carrier family 25 member 25 | 1.0 | 7.2 | 0.006 |
| Irs2 | 29376 | insulin receptor substrate 2 | 0.9 | 6.3 | 0.000 |
| Gadd45g | 291005 | growth arrest and DNA-damage-inducible, gamma | 0.9 | 4.9 | 0.004 |
| Nfil3 | 114519 | nuclear factor, interleukin 3 regulated | 0.9 | 3.7 | 0.006 |
| Rnd3 | 295588 | Rho family GTPase 3 | 0.9 | 4.5 | 0.004 |
| Midn | 314623 | midnolin | 0.9 | 5.6 | 0.000 |
| Rasl11a | 304268 | RAS-like family 11 member A | 0.9 | 1.6 | 0.012 |
| Cdkn1a | 114851 | cyclin-dependent kinase inhibitor 1A | 0.9 | 3.8 | 0.006 |
| Klf10 | 81813 | Kruppel-like factor 10 | 0.9 | 4.1 | 0.000 |
| Dusp1 | 114856 | dual specificity phosphatase 1 | 0.9 | 6.1 | 0.008 |
| Cyp2s1 | 308445 | cytochrome P450, family 2, subfamily s, polypeptide 1 | 0.9 | 3.9 | 0.006 |
| Inhba | 29200 | inhibin subunit beta A | 0.9 | 2.7 | 0.044 |
| Plk5 | 314627 | polo-like kinase 5 | 0.8 | 5.5 | 0.003 |
| Nptx2 | 288475 | neuronal pentraxin 2 | 0.8 | 5.3 | 0.003 |
| Upb1 | 116593 | beta-ureidopropionase 1 | 0.8 | 1.7 | 0.016 |
| LOC689986 | 689986 | hypothetical protein | 0.8 | 6.2 | 0.000 |
| Dnajb5 | 313811 | DnaJ heat shock protein family (Hsp40) member B5 | 0.8 | 7.3 | 0.000 |
| Krt17 | 287702 | keratin 17 | 0.8 | 4.0 | 0.050 |
| Per1 | 287422 | period circadian regulator 1 | 0.7 | 6.7 | 0.002 |
| Maff | 366960 | MAF bZIP transcription factor F | 0.7 | 1.0 | 0.024 |
| Pdp1 | 54705 | pyruvate dehydrogenase phosphatase catalytic subunit 1 | 0.7 | 7.5 | 0.000 |
| Bag3 | 293524 | BAG cochaperone 3 | 0.7 | 4.5 | 0.003 |
| Dusp2 | 311406 | dual specificity phosphatase 2 | 0.7 | 2.4 | 0.025 |
| Peli1 | 305549 | pellino E3 ubiquitin protein ligase 1 | 0.7 | 5.9 | 0.000 |
| Mir770 | 100314207 | microRNA 770 | 0.7 | 4.2 | 0.033 |
| RGD1566401 | 500717 | similar to GTL2, imprinted maternally expressed untranslated | 0.7 | 8.9 | 0.002 |
| Kif28p | 289309 | kinesin family member 28, pseudogene | 0.7 | 2.1 | 0.010 |
| Spry4 | 291610 | sprouty RTK signaling antagonist 4 | 0.7 | 3.4 | 0.026 |
| Cyp4f6 | 266689 | cytochrome P450, family 4, subfamily f, polypeptide 6 | 0.7 | 4.1 | 0.039 |
| Celsr3 | 83466 | cadherin, EGF LAG seven-pass G-type receptor 3 | 0.7 | 6.0 | 0.027 |
| Snhg11 | 362256 | small nucleolar RNA host gene 11 | 0.7 | 6.7 | 0.007 |
| B3galt2 | 686081 | Beta-1,3-galactosyltransferase 2 | 0.6 | 3.3 | 0.010 |
| Kdm6b | 363630 | lysine demethylase 6B | 0.6 | 5.4 | 0.003 |
| Pdzd7 | 293996 | PDZ domain containing 7 | 0.6 | 3.0 | 0.000 |
| Kcnip2 | 56817 | potassium voltage-gated channel interacting protein 2 | 0.6 | 2.9 | 0.013 |
| Cited2 | 114490 | Cbp/p300-interacting transactivator, with Glu/Asp-rich carboxy-terminal domain, 2 | 0.6 | 6.0 | 0.001 |
| Arhgef3 | 290541 | Rho guanine nucleotide exchange factor 3 | 0.6 | 6.6 | 0.000 |
| Clcf1 | 365395 | cardiotrophin-like cytokine factor 1 | 0.6 | 4.1 | 0.036 |
| Elmo3 | 291962 | engulfment and cell motility 3 | 0.6 | 2.6 | 0.039 |
| Lime1 | 362289 | Lck interacting transmembrane adaptor 1 | 0.6 | 4.5 | 0.007 |
| Phyhd1 | 296621 | phytanoyl-CoA dioxygenase domain containing 1 | 0.6 | 4.0 | 0.037 |
| Per2 | 63840 | period circadian regulator 2 | 0.6 | 5.9 | 0.001 |
| Kcnf1 | 298908 | potassium voltage-gated channel modifier subfamily F member 1 | 0.6 | 6.5 | 0.000 |
| Cdc42ep3 | 313838 | CDC42 effector protein 3 | 0.6 | 4.9 | 0.000 |
| Col11a2 | 294279 | collagen type XI alpha 2 chain | 0.6 | 7.6 | 0.019 |
| Ankrd61 | 689907 | ankyrin repeat domain 61 | 0.6 | 1.1 | 0.047 |
| Gpr63 | 297952 | G protein-coupled receptor 63 | 0.6 | 3.6 | 0.041 |
| Cebpb | 24253 | CCAAT/enhancer binding protein beta | 0.6 | 5.2 | 0.000 |
| Gpr22 | 298944 | G protein-coupled receptor 22 | 0.6 | 3.9 | 0.011 |
| Crem | 25620 | cAMP responsive element modulator | 0.6 | 4.6 | 0.002 |
| Frmd6 | 257646 | FERM domain containing 6 | 0.6 | 6.1 | 0.001 |
| Sdf2l1 | 680945 | stromal cell-derived factor 2-like 1 | 0.6 | 4.9 | 0.003 |
| Ppp1r15a | 171071 | protein phosphatase 1, regulatory subunit 15A | 0.6 | 5.1 | 0.031 |
| Phlda1 | 29380 | pleckstrin homology-like domain, family A, member 1 | 0.6 | 6.1 | 0.001 |
| Pard6a | 307799 | par-6 family cell polarity regulator alpha | 0.6 | 4.5 | 0.001 |
| Dot1l | 362831 | DOT1 like histone lysine methyltransferase | 0.6 | 5.8 | 0.030 |
| Lrrfip2 | 301035 | LRR binding FLII interacting protein 2 | 0.6 | 4.8 | 0.001 |
| Stk40 | 360230 | serine/threonine kinase 40 | 0.6 | 5.9 | 0.004 |
| Trank1 | 316022 | tetratricopeptide repeat and ankyrin repeat containing 1 | 0.6 | 8.1 | 0.010 |
| Htr1b | 25075 | 5-hydroxytryptamine receptor 1B | 0.6 | 5.2 | 0.013 |
| Plekhn1 | 298694 | pleckstrin homology domain containing N1 | 0.6 | 3.8 | 0.015 |
| Lpin3 | 362261 | lipin 3 | 0.6 | 5.2 | 0.004 |
| Vwa5b2 | 303812 | von Willebrand factor A domain containing 5B2 | 0.6 | 5.5 | 0.011 |
| Miat | 102552664 | myocardial infarction associated transcript | 0.6 | 5.9 | 0.021 |
| Suv420h2 | 308345 | lysine methyltransferase 5C | 0.6 | 4.8 | 0.022 |
| Cpne9 | 297516 | copine family member 9 | 0.6 | 3.3 | 0.026 |
| Acta2 | 81633 | actin alpha 2, smooth muscle | 0.6 | 4.3 | 0.002 |
| Mesdc1 | 308795 | talin rod domain containing 1 | 0.5 | 7.6 | 0.003 |
| Grasp | 192254 | trafficking regulator and scaffold protein tamalin | 0.5 | 6.7 | 0.005 |
| Bcl6 | 303836 | BCL6, transcription repressor | 0.5 | 4.2 | 0.006 |
| Jmjd6 | 360665 | jumonji domain containing 6, arginine demethylase and lysine hydroxylase | 0.5 | 4.5 | 0.008 |
| Xkr8 | 313033 | XK related 8 | 0.5 | 3.7 | 0.049 |
| Dusp14 | 360580 | dual specificity phosphatase 14 | 0.5 | 5.1 | 0.008 |
| Ahsa2 | 305577 | activator of HSP90 ATPase homolog 2 | 0.5 | 4.3 | 0.026 |
| Kcnab3 | 58981 | potassium voltage-gated channel subfamily A regulatory beta subunit 3 | 0.5 | 3.4 | 0.021 |
| Kctd17 | 300317 | potassium channel tetramerization domain containing 17 | 0.5 | 8.2 | 0.010 |
| Vgf | 29461 | VGF nerve growth factor inducible | 0.5 | 8.9 | 0.046 |
| Zdhhc13 | 365252 | zinc finger DHHC-type palmitoyltransferase 13 | 0.5 | 4.2 | 0.050 |
| Ccdc117 | 498404 | coiled-coil domain containing 117 | 0.5 | 4.1 | 0.000 |
| Dnajb1 | 361384 | DnaJ heat shock protein family (Hsp40) member B1 | 0.5 | 6.5 | 0.000 |
| Rgs2 | 84583 | regulator of G-protein signaling 2 | 0.5 | 6.6 | 0.003 |
| Hspa5 | 25617 | heat shock protein family A (Hsp70) member 5 | 0.5 | 8.6 | 0.001 |
| Ddit4 | 140942 | DNA-damage-inducible transcript 4 | 0.5 | 7.4 | 0.001 |
| Rsrp1 | 362626 | arginine and serine rich protein 1 | 0.5 | 8.3 | 0.008 |
| Zfhx2 | 305888 | zinc finger homeobox 2 | 0.5 | 6.6 | 0.029 |
| Rreb1 | 306873 | ras responsive element binding protein 1 | 0.5 | 4.2 | 0.025 |
| Irf3 | 292892 | interferon regulatory factor 3 | 0.5 | 5.1 | 0.003 |
| Foxj1 | 116557 | forkhead box J1 | 0.5 | 4.6 | 0.012 |
| Sstr2 | 54305 | somatostatin receptor 2 | 0.5 | 2.8 | 0.016 |
| Zfp692 | 303164 | zinc finger protein 692 | 0.5 | 4.8 | 0.045 |
| Slc26a10 | 366909 | solute carrier family 26, member 10 | 0.5 | 4.5 | 0.017 |
| Fcho1 | 290639 | FCH and mu domain containing endocytic adaptor 1 | 0.5 | 7.3 | 0.007 |
| Cwc25 | 360613 | CWC25 spliceosome-associated protein homolog | 0.5 | 4.5 | 0.011 |
| Serpinh1 | 29345 | serpin family H member 1 | 0.5 | 6.1 | 0.016 |
| Cbr3 | 304078 | carbonyl reductase 3 | 0.5 | 5.8 | 0.007 |
| Dlk2 | 316232 | delta like non-canonical Notch ligand 2 | 0.5 | 5.6 | 0.033 |
| Echdc2 | 298381 | enoyl CoA hydratase domain containing 2 | 0.5 | 4.7 | 0.026 |
| Agt | 24179 | angiotensinogen | 0.5 | 6.2 | 0.006 |
| Zbed3 | 361881 | zinc finger, BED-type containing 3 | 0.5 | 4.8 | 0.026 |
| Manf | 315989 | mesencephalic astrocyte-derived neurotrophic factor | 0.4 | 5.7 | 0.000 |
| Xbp1 | 289754 | X-box binding protein 1 | 0.4 | 7.0 | 0.000 |
| Adamts19 | 361332 | ADAM metallopeptidase with thrombospondin type 1 motif, 19 | 0.4 | 3.6 | 0.004 |
| Sra1 | 252891 | steroid receptor RNA activator 1 | 0.4 | 5.3 | 0.038 |
| Tsc22d3 | 83514 | TSC22 domain family, member 3 | 0.4 | 6.7 | 0.008 |
| Rps6kb2 | 361696 | ribosomal protein S6 kinase B2 | 0.4 | 5.2 | 0.012 |
| Noct | 310395 | nocturnin | 0.4 | 5.3 | 0.000 |
| Fam181b | 499205 | family with sequence similarity 181, member B | 0.4 | 5.7 | 0.032 |
| Mbd6 | 362892 | methyl-CpG binding domain protein 6 | 0.4 | 5.7 | 0.025 |
| Mroh7 | 298301 | maestro heat-like repeat family member 7 | 0.4 | 3.4 | 0.002 |
| Fam193b | 498703 | family with sequence similarity 193, member B | 0.4 | 5.9 | 0.016 |
| Eif4ebp1 | 116636 | eukaryotic translation initiation factor 4E binding protein 1 | 0.4 | 3.7 | 0.047 |
| Klf5 | 84410 | Kruppel-like factor 5 | 0.4 | 4.9 | 0.000 |
| P3h3 | 297595 | G protein-coupled receptor 162 | 0.4 | 5.6 | 0.005 |
| Clasrp | 499390 | CLK4-associating serine/arginine rich protein | 0.4 | 6.1 | 0.002 |
| Tnni3 | 29248 | troponin I3, cardiac type | 0.4 | 4.9 | 0.016 |
| Nts | 299757 | neurotensin | 0.4 | 3.7 | 0.001 |
| Wnk4 | 287715 | WNK lysine deficient protein kinase 4 | 0.4 | 6.1 | 0.036 |
| Stk38 | 361813 | serine/threonine kinase 38 | 0.4 | 5.0 | 0.016 |
| Arse | 310326 | arylsulfatase E | 0.4 | 4.8 | 0.019 |
| Mif4gd | 360659 | MIF4G domain containing | 0.4 | 4.1 | 0.011 |
| Akap8l | 299569 | A-kinase anchoring protein 8 like | 0.4 | 6.8 | 0.003 |
| L3hypdh | 314214 | trans-L-3-hydroxyproline dehydratase | 0.4 | 1.7 | 0.049 |
| Gipr | 25024 | gastric inhibitory polypeptide receptor | 0.4 | 3.5 | 0.049 |
| Rnf217 | 292188 | ring finger protein 217 | 0.4 | 3.8 | 0.048 |
| Panx1 | 315435 | Pannexin 1 | 0.4 | 5.1 | 0.038 |
| Amt | 306586 | aminomethyltransferase | 0.4 | 5.1 | 0.005 |
| Prpf38b | 499691 | pre-mRNA processing factor 38B | 0.4 | 6.1 | 0.029 |
| Mafk | 246760 | MAF bZIP transcription factor K | 0.4 | 4.5 | 0.025 |
| Tmem132e | 287564 | transmembrane protein 132E | 0.4 | 6.1 | 0.007 |
| Matk | 60450 | megakaryocyte-associated tyrosine kinase | 0.4 | 7.5 | 0.003 |
| Myh11 | 24582 | myosin heavy chain 11 | 0.4 | 4.4 | 0.016 |
| LOC500300 | 500300 | autophagy regulator | 0.4 | 3.5 | 0.037 |
| Rgl2 | 294283 | ral guanine nucleotide dissociation stimulator-like 2 | 0.4 | 5.8 | 0.003 |
| Car11 | 308588 | carbonic anhydrase 11 | 0.4 | 8.9 | 0.003 |
| Slc12a3 | 54300 | solute carrier family 12 member 3 | 0.4 | 2.1 | 0.027 |
| Ano6 | 315272 | anoctamin 6 | 0.4 | 4.5 | 0.006 |
| Aifm3 | 303786 | apoptosis inducing factor, mitochondria associated 3 | 0.4 | 6.0 | 0.008 |
| Pask | 301617 | PAS domain containing serine/threonine kinase | 0.4 | 3.7 | 0.040 |
| Jun | 24516 | Jun proto-oncogene, AP-1 transcription factor subunit | 0.4 | 6.1 | 0.002 |
| Vegfa | 83785 | vascular endothelial growth factor A | 0.4 | 7.4 | 0.018 |
| Srxn1 | 296271 | sulfiredoxin 1 | 0.4 | 6.2 | 0.026 |
| Fmnl1 | 287746 | formin-like 1 | 0.4 | 7.8 | 0.031 |
| Gpr19 | 312787 | G protein-coupled receptor 19 | 0.4 | 3.5 | 0.031 |
| Ttll3 | 362415 | tubulin tyrosine ligase like 3 | 0.4 | 3.6 | 0.014 |
| LOC688452 | 688452 | sperm acrosome associated 6 | 0.4 | 3.7 | 0.027 |
| Smarcd2 | 83833 | SWI/SNF related, matrix associated, actin dependent regulator of chromatin, subfamily d, member 2 | 0.4 | 5.0 | 0.039 |
| Hdac10 | 362981 | histone deacetylase 10 | 0.4 | 4.3 | 0.011 |
| Chordc1 | 315447 | cysteine and histidine rich domain containing 1 | 0.4 | 5.5 | 0.003 |
| Ikbke | 363984 | inhibitor of nuclear factor kappa B kinase subunit epsilon | 0.4 | 1.5 | 0.032 |
| Smad7 | 81516 | SMAD family member 7 | 0.4 | 6.2 | 0.038 |
| P4ha2 | 360526 | prolyl 4-hydroxylase subunit alpha 2 | 0.4 | 4.5 | 0.013 |
| Nxf1 | 59087 | nuclear RNA export factor 1 | 0.4 | 6.3 | 0.023 |
| Tmem208 | 291963 | transmembrane protein 208 | 0.4 | 5.2 | 0.025 |
| Etv5 | 303828 | ETS variant transcription factor 5 | 0.4 | 6.7 | 0.011 |
| Crtc2 | 310615 | CREB regulated transcription coactivator 2 | 0.4 | 5.4 | 0.050 |
| Abcd4 | 299196 | ATP binding cassette subfamily D member 4 | 0.4 | 3.6 | 0.003 |
| Srf | 501099 | serum response factor | 0.3 | 7.5 | 0.004 |
| Zfp385a | 685474 | zinc finger protein 385A | 0.3 | 6.9 | 0.025 |
| Slc19a3 | 316559 | solute carrier family 19 member 3 | 0.3 | 2.6 | 0.010 |
| Pnpla7 | 246246 | patatin-like phospholipase domain containing 7 | 0.3 | 4.9 | 0.027 |
| Hrh3 | 85268 | histamine receptor H3 | 0.3 | 9.3 | 0.019 |
| Cebpd | 25695 | CCAAT/enhancer binding protein delta | 0.3 | 3.7 | 0.026 |
| Ptpn1 | 24697 | protein tyrosine phosphatase, non-receptor type 1 | 0.3 | 5.0 | 0.025 |
| Ube2s | 292588 | ubiquitin-conjugating enzyme E2S | 0.3 | 7.2 | 0.017 |
| Cnr1 | 25248 | cannabinoid receptor 1 | 0.3 | 8.5 | 0.020 |
| G0s2 | 289388 | G0/G1switch 2 | 0.3 | 3.3 | 0.045 |
| Myl9 | 296313 | myosin light chain 9 | 0.3 | 4.5 | 0.016 |
| Kcnt1 | 60444 | potassium sodium-activated channel subfamily T member 1 | 0.3 | 7.5 | 0.002 |
| Fam57b | 293493 | TLC domain containing 3B | 0.3 | 4.4 | 0.011 |
| Rhot2 | 287156 | ras homolog family member T2 | 0.3 | 6.6 | 0.050 |
| Tagln | 25123 | transgelin | 0.3 | 4.8 | 0.002 |
| Atxn2l | 361649 | ataxin 2-like | 0.3 | 7.3 | 0.031 |
| Zfp384 | 171018 | zinc finger protein 384 | 0.3 | 5.5 | 0.039 |
| Pkd1 | 24650 | polycystin 1, transient receptor potential channel interacting | 0.3 | 7.6 | 0.045 |
| Ankrd10 | 361183 | ankyrin repeat domain 10 | 0.3 | 5.6 | 0.006 |
| Rprml | 685826 | reprimo-like | 0.3 | 7.1 | 0.003 |
| Emd | 25437 | emerin | 0.3 | 4.7 | 0.009 |
| Adamts10 | 314655 | ADAM metallopeptidase with thrombospondin type 1 motif, 10 | 0.3 | 4.8 | 0.032 |
| Rheb | 26954 | Ras homolog, mTORC1 binding | 0.3 | 7.2 | 0.000 |
| Sf1 | 117855 | splicing factor 1 | 0.3 | 7.9 | 0.003 |
| Hapln2 | 64057 | hyaluronan and proteoglycan link protein 2 | 0.3 | 7.2 | 0.049 |
| Acy1 | 300981 | aminoacylase 1 | 0.3 | 5.2 | 0.036 |
| Dmkn | 361548 | dermokine | 0.3 | 6.0 | 0.035 |
| Wfdc2 | 286888 | WAP four-disulfide core domain 2 | 0.3 | 3.9 | 0.031 |
| Cdc42ep4 | 303653 | CDC42 effector protein 4 | 0.3 | 7.2 | 0.028 |
| Coq10b | 301416 | coenzyme Q10B | 0.3 | 4.5 | 0.041 |
| Ccnd3 | 25193 | cyclin D3 | 0.3 | 5.6 | 0.021 |
| Gpt | 81670 | glutamic--pyruvic transaminase | 0.3 | 5.5 | 0.027 |
| Arhgap4 | 246249 | Rho GTPase activating protein 4 | 0.3 | 3.3 | 0.037 |
| Chchd10 | 361824 | coiled-coil-helix-coiled-coil-helix domain containing 10 | 0.3 | 9.2 | 0.012 |
| Csf2ra | 652957 | colony stimulating factor 2 receptor subunit alpha | 0.3 | 3.2 | 0.033 |
| Xrcc1 | 84495 | X-ray repair cross complementing 1 | 0.3 | 6.1 | 0.010 |
| Kcna5 | 25470 | potassium voltage-gated channel subfamily A member 5 | 0.3 | 5.2 | 0.044 |
| Fkbp4 | 260321 | FKBP prolyl isomerase 4 | 0.3 | 7.9 | 0.006 |
| Trak1 | 316085 | trafficking kinesin protein 1 | 0.3 | 7.2 | 0.001 |
| Dennd6b | 362983 | DENN domain containing 6B | 0.3 | 5.3 | 0.016 |
| Mfsd10 | 305449 | major facilitator superfamily domain containing 10 | 0.3 | 4.7 | 0.012 |
| Gpld1 | 291132 | glycosylphosphatidylinositol specific phospholipase D1 | 0.3 | 5.3 | 0.023 |
| Zfp958 | 100302405 | zinc finger protein 958 | 0.3 | 3.6 | 0.027 |
| Pagr1 | 293500 | Paxip1-associated glutamate-rich protein 1 | 0.3 | 5.0 | 0.027 |
| Cys1 | 690489 | cystin 1 | 0.3 | 4.2 | 0.002 |
| Plekhh3 | 360634 | pleckstrin homology, MyTH4 and FERM domain containing H3 | 0.3 | 5.0 | 0.049 |
| Nmu | 63887 | neuromedin U | 0.3 | 1.6 | 0.045 |
| Snrnp70 | 361574 | small nuclear ribonucleoprotein U1 subunit 70 | 0.3 | 7.3 | 0.009 |
| Lrrc73 | 501101 | leucine rich repeat containing 73 | 0.3 | 5.9 | 0.037 |
| Taf10 | 293345 | TATA-box binding protein associated factor 10 | 0.3 | 6.5 | 0.026 |
| Slc15a4 | 246280 | solute carrier family 15 member 4 | 0.3 | 5.1 | 0.048 |
| Atp13a1 | 290673 | ATPase 13A1 | 0.3 | 5.9 | 0.019 |
| Lin7b | 60377 | lin-7 homolog B, crumbs cell polarity complex component | 0.3 | 7.6 | 0.010 |
| Spred1 | 296072 | sprouty-related, EVH1 domain containing 1 | 0.3 | 5.3 | 0.015 |
| Emid1 | 685462 | EMI domain containing 1 | 0.3 | 5.6 | 0.017 |
| Atp6v0e1 | 94170 | ATPase H+ transporting V0 subunit e1 | 0.3 | 5.4 | 0.010 |
| Pdcd6 | 308061 | programmed cell death 6 | 0.3 | 5.8 | 0.026 |
| Josd1 | 315134 | Josephin domain containing 1 | 0.3 | 6.8 | 0.000 |
| Baz1b | 368002 | bromodomain adjacent to zinc finger domain, 1B | 0.3 | 7.8 | 0.022 |
| Atp5g1 | 29754 | ATP synthase membrane subunit c locus 1 | 0.3 | 8.2 | 0.039 |
| Cry1 | 299691 | cryptochrome circadian regulator 1 | 0.3 | 5.5 | 0.027 |
| Dnajb11 | 360734 | DnaJ heat shock protein family (Hsp40) member B11 | 0.3 | 6.1 | 0.003 |
| Dexi | 497857 | Dexi homolog | 0.3 | 6.0 | 0.038 |
| Atg4d | 686505 | autophagy related 4D, cysteine peptidase | 0.3 | 5.5 | 0.029 |
| Nrg2 | 432361 | neuregulin 2 | 0.3 | 4.8 | 0.029 |
| Spns1 | 361648 | sphingolipid transporter 1 | 0.3 | 7.2 | 0.004 |
| Sfpq | 252855 | splicing factor proline and glutamine rich | 0.3 | 7.2 | 0.004 |
| Inpp5j | 171088 | inositol polyphosphate-5-phosphatase J | 0.3 | 6.1 | 0.041 |
| Clk3 | 171305 | CDC-like kinase 3 | 0.3 | 6.0 | 0.037 |
| Pld2 | 25097 | phospholipase D2 | 0.3 | 5.9 | 0.010 |
| Ppdpf | 296470 | pancreatic progenitor cell differentiation and proliferation factor | 0.3 | 6.4 | 0.049 |
| Hdac5 | 84580 | histone deacetylase 5 | 0.3 | 7.7 | 0.020 |
| Spata2L | 498963 | spermatogenesis associated 2-like | 0.3 | 7.6 | 0.022 |
| Repin1 | 445541 | replication initiator 1 | 0.3 | 6.0 | 0.003 |
| Gramd1b | 300644 | GRAM domain containing 1B | 0.3 | 5.4 | 0.027 |
| Pabpn1 | 116697 | poly(A) binding protein, nuclear 1 | 0.3 | 7.6 | 0.034 |
| Igsf9 | 304982 | immunoglobulin superfamily, member 9 | 0.3 | 3.5 | 0.026 |
| Pqlc1 | 361352 | solute carrier family 66 member 2 | 0.3 | 5.3 | 0.015 |
| Rbm14 | 170900 | RNA binding motif protein 14 | 0.3 | 5.6 | 0.003 |
| Col16a1 | 366474 | collagen type XVI alpha 1 chain | 0.3 | 4.7 | 0.043 |
| Dpp7 | 83799 | dipeptidylpeptidase 7 | 0.3 | 5.8 | 0.007 |
| P4ha1 | 64475 | prolyl 4-hydroxylase subunit alpha 1 | 0.3 | 5.6 | 0.002 |
| Iqgap3 | 310621 | IQ motif containing GTPase activating protein 3 | 0.3 | 5.3 | 0.034 |
| Dph1 | 287523 | diphthamide biosynthesis 1 | 0.3 | 5.1 | 0.000 |
| Rcor1 | 102554884 | REST corepressor 1 | 0.3 | 4.6 | 0.010 |
| Acin1 | 305884 | apoptotic chromatin condensation inducer 1 | 0.3 | 7.5 | 0.046 |
| Ddx17 | 315133 | DEAD box helicase 17 | 0.3 | 7.5 | 0.008 |
| Ddx39b | 114612 | DExD-box helicase 39B | 0.3 | 7.9 | 0.008 |
| Stip1 | 192277 | stress-induced phosphoprotein 1 | 0.3 | 7.8 | 0.007 |
| Bcat2 | 64203 | branched chain amino acid transaminase 2 | 0.3 | 3.9 | 0.039 |
| Samd4b | 308473 | sterile alpha motif domain containing 4B | 0.3 | 6.3 | 0.032 |
| Klc4 | 316226 | kinesin light chain 4 | 0.3 | 5.4 | 0.016 |
| Leng8 | 361506 | leukocyte receptor cluster member 8 | 0.3 | 7.5 | 0.007 |
| Otof | 84573 | otoferlin | 0.2 | 6.1 | 0.050 |
| Cfap20 | 307642 | cilia and flagella associated protein 20 | 0.2 | 6.2 | 0.003 |
| Slc22a3 | 29504 | solute carrier family 22 member 3 | 0.2 | 4.8 | 0.032 |
| Gatsl2 | 304410 | ytosolic arginine sensor for mTORC1 subunit 2 | 0.2 | 7.5 | 0.026 |
| Sppl2b | 362828 | signal peptide peptidase-like 2B | 0.2 | 6.2 | 0.032 |
| Mzf1 | 361508 | myeloid zinc finger 1 | 0.2 | 5.6 | 0.007 |
| Zfp775 | 312309 | zinc finger protein 775 | 0.2 | 4.6 | 0.041 |
| Ncoa5 | 296372 | nuclear receptor coactivator 5 | 0.2 | 6.6 | 0.007 |
| Fam98c | 292764 | family with sequence similarity 98, member C | 0.2 | 6.0 | 0.031 |
| Cx3cl1 | 89808 | C-X3-C motif chemokine ligand 1 | 0.2 | 10.1 | 0.031 |
| Mest | 58827 | mesoderm specific transcript | 0.2 | 4.4 | 0.027 |
| Ndufs7 | 362837 | NADH:ubiquinone oxidoreductase core subunit S7 | 0.2 | 7.7 | 0.045 |
| Slc25a33 | 691431 | solute carrier family 25 member 33 | 0.2 | 6.0 | 0.014 |
| Syt6 | 60565 | synaptotagmin 6 | 0.2 | 5.4 | 0.046 |
| Top3b | 287930 | DNA topoisomerase III beta | 0.2 | 5.5 | 0.048 |
| F8 | 302470 | coagulation factor VIII | 0.2 | 5.3 | 0.020 |
| Hcfc1r1 | 287097 | host cell factor C1 regulator 1 | 0.2 | 7.0 | 0.045 |
| Zdhhc2 | 246326 | zinc finger DHHC-type palmitoyltransferase 2 | 0.2 | 6.0 | 0.027 |
| Lrg1 | 367455 | leucine-rich alpha-2-glycoprotein 1 | 0.2 | 3.4 | 0.017 |
| Hsd17b8 | 361802 | hydroxysteroid (17-beta) dehydrogenase 8 | 0.2 | 5.5 | 0.040 |
| Creld2 | 362978 | cysteine-rich with EGF-like domains 2 | 0.2 | 4.5 | 0.015 |
| Ust | 361450 | uronyl-2-sulfotransferase | 0.2 | 4.6 | 0.045 |
| Rcan2 | 140666 | regulator of calcineurin 2 | 0.2 | 9.0 | 0.031 |
| Polrmt | 299604 | RNA polymerase mitochondrial | 0.2 | 5.3 | 0.033 |
| Efnb1 | 25186 | ephrin B1 | 0.2 | 4.3 | 0.041 |
| Pnn | 368070 | pinin, desmosome associated protein | 0.2 | 6.2 | 0.028 |
| Nme3 | 85269 | NME/NM23 nucleoside diphosphate kinase 3 | 0.2 | 6.2 | 0.017 |
| Arntl | 29657 | aryl hydrocarbon receptor nuclear translocator-like | 0.2 | 5.5 | 0.005 |
| Pdia4 | 116598 | protein disulfide isomerase family A, member 4 | 0.2 | 5.7 | 0.012 |
| Rnf112 | 24916 | ring finger protein 112 | 0.2 | 7.7 | 0.003 |
| Bcar3 | 310838 | BCAR3 adaptor protein, NSP family member | 0.2 | 5.7 | 0.033 |
| Heca | 308624 | hdc homolog, cell cycle regulator | 0.2 | 5.3 | 0.027 |
| Inha | 24504 | inhibin subunit alpha | 0.2 | 3.6 | 0.032 |
| Tmem109 | 361732 | transmembrane protein 109 | 0.2 | 5.8 | 0.043 |
| Ptms | 83801 | parathymosin | 0.2 | 11.3 | 0.038 |
| Fnbp4 | 311183 | formin binding protein 4 | 0.2 | 5.3 | 0.033 |
| Aldh1l1 | 64392 | aldehyde dehydrogenase 1 family, member L1 | 0.2 | 4.5 | 0.045 |
| Dnpep | 301529 | aspartyl aminopeptidase | 0.2 | 7.2 | 0.007 |
| Abcb6 | 140669 | ATP binding cassette subfamily B member 6 | 0.2 | 6.0 | 0.049 |
| RGD1560394 | 289728 | RGD1560394 | 0.2 | 5.8 | 0.045 |
| Pitpnm2 | 304474 | phosphatidylinositol transfer protein, membrane-associated 2 | 0.2 | 9.0 | 0.034 |
| Pdyn | 29190 | prodynorphin | 0.2 | 8.6 | 0.003 |
| L3mbtl2 | 300320 | L3MBTL histone methyl-lysine binding protein 2 | 0.2 | 6.1 | 0.029 |
| Mapk1ip1 | 499280 | mitogen-activated protein kinase 1 interacting protein 1 | 0.2 | 5.1 | 0.025 |
| Arpp21 | 363153 | cAMP regulated phosphoprotein 21 | 0.2 | 10.9 | 0.033 |
| Dgka | 140866 | diacylglycerol kinase, alpha | 0.2 | 5.8 | 0.032 |
| Dusp26 | 306527 | dual specificity phosphatase 26 | 0.2 | 6.9 | 0.039 |
| Sdccag3 | 306322 | endosome associated trafficking regulator 1 | 0.2 | 6.1 | 0.015 |
| Pcsk7 | 29606 | proprotein convertase subtilisin/kexin type 7 | 0.2 | 4.9 | 0.041 |
| Begain | 79146 | brain-enriched guanylate kinase-associated | 0.2 | 8.3 | 0.021 |
| Rabac1 | 83583 | Rab acceptor 1 | 0.2 | 8.4 | 0.036 |
| Pfas | 287420 | phosphoribosylformylglycinamidine synthase | 0.2 | 5.9 | 0.039 |
| Acadvl | 25363 | acyl-CoA dehydrogenase, very long chain | 0.2 | 6.1 | 0.029 |
| Srsf11 | 502603 | serine and arginine rich splicing factor 11 | 0.2 | 6.6 | 0.047 |
| Dtx3 | 500847 | deltex E3 ubiquitin ligase 3 | 0.2 | 7.5 | 0.040 |
| RGD1304567 | 362671 | similar to RIKEN cDNA A430005L14 | 0.2 | 5.0 | 0.039 |
| Rab24 | 361208 | Max dimerization protein 3 | 0.2 | 6.6 | 0.024 |
| Dnaja1 | 65028 | DnaJ heat shock protein family (Hsp40) member A1 | 0.2 | 8.2 | 0.003 |
| RGD1311739 | 311428 | similar to RIKEN cDNA 1700037H04 | 0.2 | 8.0 | 0.027 |
| Unc45b | 303373 | unc-45 myosin chaperone B | 0.2 | 2.2 | 0.032 |
| Mnt | 287521 | MAX network transcriptional repressor | 0.2 | 5.9 | 0.016 |
| Gba2 | 298399 | glucosylceramidase beta 2 | 0.2 | 6.6 | 0.014 |
| Klhl29 | 298867 | kelch-like family member 29 | 0.2 | 6.4 | 0.037 |
| Grik5 | 24407 | glutamate ionotropic receptor kainate type subunit 5 | 0.2 | 9.0 | 0.024 |
| Kifc2 | 300053 | kinesin family member C2 | 0.2 | 8.3 | 0.031 |
| Ahsa1 | 681996 | activator of Hsp90 ATPase activity 1 | 0.2 | 7.3 | 0.037 |
| Gigyf1 | 304378 | GRB10 interacting GYF protein 1 | 0.2 | 5.8 | 0.010 |
| Bag1 | 297994 | BAG cochaperone 1 | 0.2 | 6.7 | 0.038 |
| Ahnak | 191572 | AHNAK nucleoprotein | 0.2 | 6.1 | 0.039 |
| Rilpl2 | 288652 | Rab interacting lysosomal protein-like 2 | 0.2 | 6.0 | 0.024 |
| Herpud1 | 85430 | homocysteine inducible ER protein with ubiquitin like domain 1 | 0.2 | 5.9 | 0.002 |
| Tob2 | 315159 | transducer of ERBB2, 2 | 0.2 | 5.1 | 0.026 |
| Actn1 | 81634 | actinin, alpha 1 | 0.2 | 8.4 | 0.045 |
| Fam220a | 498145 | family with sequence similarity 220, member A | 0.2 | 4.6 | 0.011 |
| Gcdh | 364975 | glutaryl-CoA dehydrogenase | 0.2 | 6.3 | 0.021 |
| Sertad4 | 360899 | SERTA domain containing 4 | 0.2 | 6.1 | 0.037 |
| Dgkq | 100361138 | diacylglycerol kinase, theta | 0.2 | 5.4 | 0.046 |
| Iffo1 | 362437 | intermediate filament family orphan 1 | 0.2 | 7.1 | 0.024 |
| Fbxo44 | 500587 | F-box protein 44 | 0.2 | 7.5 | 0.032 |
| Taf15 | 287571 | TATA-box binding protein associated factor 15 | 0.2 | 6.3 | 0.037 |
| Zcchc7 | 298086 | zinc finger CCHC-type containing 7 | 0.2 | 4.6 | 0.011 |
| Iqub | 296936 | IQ motif and ubiquitin domain containing | 0.2 | 4.2 | 0.049 |
| Zbtb17 | 313666 | zinc finger and BTB domain containing 17 | 0.2 | 6.0 | 0.025 |
| Fbxw8 | 304522 | F-box and WD repeat domain containing 8 | 0.2 | 6.6 | 0.022 |
| Rbm4 | 293663 | RNA binding motif protein 4 | 0.2 | 5.3 | 0.032 |
| Klhdc8b | 306589 | kelch domain containing 8B | 0.2 | 5.7 | 0.015 |
| Fam174a | 301634 | family with sequence similarity 174, member A | 0.2 | 5.8 | 0.041 |
| Chtf8 | 364996 | chromosome transmission fidelity factor 8 | 0.2 | 7.1 | 0.011 |
| Vps9d1 | 307923 | VPS9 domain containing 1 | 0.2 | 5.5 | 0.017 |
| Abcc10 | 316231 | ATP binding cassette subfamily C member 10 | 0.2 | 4.5 | 0.033 |
| Coro7 | 192276 | coronin 7 | 0.2 | 7.9 | 0.033 |
| Mchr1 | 83567 | melanin-concentrating hormone receptor 1 | 0.2 | 4.6 | 0.004 |
| Wdr45b | 360682 | WD repeat domain 45B | 0.2 | 4.9 | 0.031 |
| Gramd1a | 361550 | GRAM domain containing 1A | 0.2 | 6.7 | 0.024 |
| Commd4 | 363068 | COMM domain containing 4 | 0.2 | 6.3 | 0.030 |
| Clvs1 | 366311 | clavesin 1 | 0.2 | 6.0 | 0.014 |
| B4galt3 | 494342 | beta-1,4-galactosyltransferase 3 | 0.2 | 5.9 | 0.025 |
| Nosip | 292894 | nitric oxide synthase interacting protein | 0.2 | 5.6 | 0.048 |
| Glcci1 | 296884 | glucocorticoid induced 1 | 0.2 | 5.2 | 0.011 |
| Ahctf1 | 360886 | AT hook containing transcription factor 1 | 0.2 | 5.9 | 0.034 |
| Dmtn | 361069 | dematin actin binding protein | 0.1 | 8.7 | 0.026 |
| Kctd1 | 291772 | potassium channel tetramerization domain containing 1 | 0.1 | 7.8 | 0.022 |
| Ifrd2 | 300994 | interferon-related developmental regulator 2 | 0.1 | 4.2 | 0.032 |
| Rgs9 | 29481 | regulator of G-protein signaling 9 | 0.1 | 9.7 | 0.004 |
| Kif20a | 361308 | kinesin family member 20A | 0.1 | 3.7 | 0.035 |
| Tomm20 | 266601 | translocase of outer mitochondrial membrane 20 | 0.1 | 8.1 | 0.005 |
| Plekhj1 | 314634 | pleckstrin homology domain containing J1 | 0.1 | 6.3 | 0.012 |
| Ttc39b | 298186 | tetratricopeptide repeat domain 39B | 0.1 | 3.8 | 0.009 |
| Dus3l | 301122 | dihydrouridine synthase 3-like | 0.1 | 5.2 | 0.015 |
| Srsf3 | 361814 | serine and arginine rich splicing factor 3 | 0.1 | 7.4 | 0.011 |
| Cep41 | 500069 | centrosomal protein 41 | 0.1 | 3.5 | 0.025 |
| Rgs8 | 54297 | regulator of G-protein signaling 8 | 0.1 | 7.7 | 0.025 |
| Myo9b | 25486 | myosin IXb | 0.1 | 6.9 | 0.029 |
| Dnajb4 | 295549 | DnaJ heat shock protein family (Hsp40) member B4 | 0.1 | 5.5 | 0.035 |
| Ppp2r3b | 682033 | protein phosphatase 2, regulatory subunit B'', beta | 0.1 | 4.1 | 0.016 |
| Mtmr4 | 287607 | myotubularin related protein 4 | 0.1 | 6.6 | 0.014 |
| Aurkc | 292554 | aurora kinase C | 0.1 | 3.5 | 0.033 |
| Clk4 | 287269 | CDC-like kinase 4 | 0.1 | 4.8 | 0.021 |
| Sncb | 113893 | synuclein, beta | 0.1 | 8.2 | 0.025 |
| Tsc22d1 | 498545 | TSC22 domain family, member 1 | 0.1 | 9.0 | 0.011 |
| Pja1 | 683077 | praja ring finger ubiquitin ligase 1 | 0.1 | 8.4 | 0.009 |
| Smim3 | 286910 | small integral membrane protein 3 | 0.1 | 4.4 | 0.008 |
| Gtf2ird1 | 246770 | GTF2I repeat domain containing 1 | 0.1 | 4.9 | 0.026 |
| Fam227a | 300074 | family with sequence similarity 227, member A | 0.1 | 3.6 | 0.030 |
| Wee1 | 308937 | WEE1 G2 checkpoint kinase | 0.1 | 5.8 | 0.035 |
| Nrbp2 | 680451 | nuclear receptor binding protein 2 | 0.1 | 7.2 | 0.005 |
| Crym | 117024 | crystallin, mu | 0.1 | 8.1 | 0.007 |
| Plcxd2 | 363781 | phosphatidylinositol-specific phospholipase C, X domain containing 2 | 0.1 | 3.8 | 0.036 |
| Gsto2 | 309465 | glutathione S-transferase omega 2 | 0.1 | 3.3 | 0.026 |
| Pcp4l1 | 685448 | Purkinje cell protein 4-like 1 | 0.1 | 8.7 | 0.015 |
| Ovca2 | 497954 | OVCA2 serine hydrolase domain containing | 0.1 | 4.0 | 0.032 |
| G6pd | 24377 | glucose-6-phosphate dehydrogenas | 0.1 | 6.4 | 0.042 |
| Trmt1 | 288914 | tRNA methyltransferase 1 | 0.1 | 5.9 | 0.022 |
| Golph3 | 78961 | golgi phosphoprotein 3 | 0.1 | 6.3 | 0.017 |
| Nus1 | 294400 | NUS1 dehydrodolichyl diphosphate synthase subunit | 0.1 | 7.0 | 0.021 |
| Pten | 50557 | phosphatase and tensin homolog | 0.1 | 5.3 | 0.009 |
| Tollip | 361677 | toll interacting protein | 0.1 | 7.3 | 0.027 |
| Angptl4 | 362850 | angiopoietin-like 4 | 0.1 | 3.3 | 0.007 |
| Reep2 | 682105 | receptor accessory protein 2 | 0.1 | 7.5 | 0.039 |
| Agap3 | 362300 | ArfGAP with GTPase domain, ankyrin repeat and PH domain 3 | 0.1 | 8.9 | 0.045 |
| Ctxn1 | 29145 | cortexin 1 | 0.1 | 10.5 | 0.041 |
| Myh14 | 308572 | myosin heavy chain 14 | 0.1 | 6.6 | 0.025 |
| Rph3a | 171039 | rabphilin 3A | 0.1 | 8.2 | 0.050 |
| Trmt2a | 287953 | tRNA methyltransferase 2 homolog A | 0.1 | 4.7 | 0.025 |
| Slc37a4 | 29573 | solute carrier family 37 member 4 | 0.1 | 5.5 | 0.008 |
| Icam5 | 313785 | intercellular adhesion molecule 5 | 0.1 | 8.6 | 0.002 |
| Ext1 | 299907 | exostosin glycosyltransferase 1 | 0.1 | 4.5 | 0.015 |
| Rasgrp2 | 361714 | RAS guanyl releasing protein 2 | 0.1 | 8.0 | 0.003 |
| Rogdi | 287061 | rogdi atypical leucine zipper | 0.1 | 7.9 | 0.046 |
| Ltk | 311337 | leukocyte receptor tyrosine kinase | 0.1 | 4.7 | 0.015 |
| Cdh6 | 25409 | cadherin 6 | 0.1 | 4.7 | 0.030 |
| Mxi1 | 25701 | MAX interactor 1, dimerization protein | 0.1 | 7.7 | 0.013 |
| Cog2 | 690961 | component of oligomeric golgi complex 2 | 0.1 | 5.5 | 0.049 |
| Rhbdl3 | 287556 | rhomboid like 3 | 0.1 | 6.4 | 0.026 |
| Cul9 | 316228 | cullin 9 | 0.1 | 7.7 | 0.049 |
| Ube2b | 81816 | ubiquitin-conjugating enzyme E2B | 0.1 | 7.5 | 0.025 |
| LOC680039 | 680039 | hypothetical protein | 0.1 | 7.8 | 0.039 |
| Rap1gap | 313644 | Rap1 GTPase-activating protein | 0.1 | 10.1 | 0.010 |
| Man2c1 | 246136 | mannosidase, alpha, class 2C, member 1 | 0.1 | 6.2 | 0.035 |
| Kitlg | 60427 | KIT ligand | 0.1 | 5.7 | 0.048 |
| Setd4 | 245975 | SET domain containing 4 | 0.1 | 3.9 | 0.035 |
| Camkv | 79011 | CaM kinase-like vesicle-associated | 0.1 | 10.5 | 0.014 |
| Caly | 192349 | calcyon neuron-specific vesicular protein | 0.1 | 8.7 | 0.013 |
| Abcb9 | 63886 | ATP binding cassette subfamily B member 9 | 0.1 | 6.6 | 0.026 |
| Mpnd | 681944 | MPN domain containing | 0.1 | 5.8 | 0.026 |
| Arpc5 | 360854 | actin related protein 2/3 complex, subunit 5 | 0.1 | 7.2 | 0.016 |
| Ostc | 362040 | oligosaccharyltransferase complex non-catalytic subunit | 0.1 | 5.4 | 0.043 |
| Prrt2 | 361651 | proline-rich transmembrane protein 2 | 0.1 | 8.1 | 0.004 |
| Plekha5 | 246237 | pleckstrin homology domain containing A5 | 0.1 | 7.0 | 0.025 |
| Cpt1c | 308579 | carnitine palmitoyltransferase 1c | 0.1 | 6.4 | 0.024 |
| Slc4a3 | 24781 | solute carrier family 4 member 3 | 0.1 | 8.2 | 0.030 |
| Galnt14 | 313878 | polypeptide N-acetylgalactosaminyltransferase 14 | 0.1 | 4.1 | 0.017 |
| Ociad2 | 100361733 | OCIA domain containing 2 | 0.1 | 5.8 | 0.011 |
| Naa30 | 498489 | N(alpha)-acetyltransferase 30, NatC catalytic subunit | 0.1 | 5.9 | 0.026 |
| Tmem180 | 309454 | major facilitator superfamily domain containing 13A | 0.1 | 5.9 | 0.049 |
| Sema7a | 315711 | semaphorin 7A (John Milton Hagen blood group) | 0.1 | 8.2 | 0.049 |
| Mmd | 303439 | monocyte to macrophage differentiation-associated | 0.1 | 8.1 | 0.042 |
| Elmo1 | 361251 | engulfment and cell motility 1 | 0.1 | 7.5 | 0.046 |
| Mtus1 | 306487 | microtubule associated scaffold protein 1 | 0.1 | 6.6 | 0.007 |
| Kcns2 | 66022 | potassium voltage-gated channel, modifier subfamily S, member 2 | 0.1 | 4.7 | 0.001 |
| Ace | 24310 | angiotensin I converting enzyme | 0.1 | 6.8 | 0.010 |
| Cacybp | 289144 | calcyclin binding protein | 0.1 | 6.6 | 0.015 |
| Man2b1 | 361378 | mannosidase, alpha, class 2B, member 1 | 0.1 | 5.4 | 0.015 |
| Pim2 | 317366 | Pim-2 proto-oncogene, serine/threonine kinase | 0.1 | 5.6 | 0.050 |
| Fbxo33 | 314157 | F-box protein 33 | 0.1 | 5.4 | 0.010 |
| Ctnnal1 | 298019 | catenin alpha-like 1 | 0.1 | 5.3 | 0.003 |
| Actr3b | 362298 | actin related protein 3B | 0.1 | 6.6 | 0.026 |
| Adck4 | 308453 | coenzyme Q8B | 0.1 | 5.7 | 0.045 |
| Wbscr17 | 288611 | polypeptide N-acetylgalactosaminyltransferase 17 | 0.1 | 4.8 | 0.001 |
| Fam178a | 499360 | SMC5-SMC6 complex localization factor 2 | 0.1 | 5.9 | 0.017 |
| Syt4 | 64440 | synaptotagmin 4 | 0.1 | 7.7 | 0.001 |
| Ube2d3 | 81920 | ubiquitin-conjugating enzyme E2D 3 | 0.1 | 7.5 | 0.031 |
| Efhd1 | 501181 | EF-hand domain family, member D1 | 0.1 | 5.2 | 0.017 |
| Hspa4 | 266759 | heat shock protein family A (Hsp70) member 4 | 0.1 | 8.1 | 0.042 |
| Elovl1 | 679532 | ELOVL fatty acid elongase 1 | 0.0 | 5.9 | 0.031 |
| Banp | 292064 | Btg3 associated nuclear protein | 0.0 | 5.1 | 0.031 |
| Mtmr2 | 315422 | myotubularin related protein 2 | 0.0 | 6.5 | 0.043 |
| Cep85 | 362622 | centrosomal protein 85 | 0.0 | 4.7 | 0.048 |
| Fn3k | 498034 | fructosamine 3 kinase | 0.0 | 4.9 | 0.013 |
| Ache | 83817 | acetylcholinesterase | 0.0 | 7.1 | 0.016 |
| Csad | 60356 | cysteine sulfinic acid decarboxylase | 0.0 | 5.6 | 0.010 |
| Il34 | 498951 | interleukin 34 | 0.0 | 6.0 | 0.034 |
| Ypel4 | 502643 | yippee-like 4 | 0.0 | 4.7 | 0.039 |
| Arl5a | 117050 | ADP-ribosylation factor like GTPase 5A | 0.0 | 4.6 | 0.004 |
| Ctsa | 296370 | cathepsin A | 0.0 | 7.4 | 0.020 |
| Hook2 | 304669 | hook microtubule-tethering protein 2 | 0.0 | 3.9 | 0.016 |
| Pnisr | 297942 | PNN interacting serine and arginine rich protein | 0.0 | 6.8 | 0.003 |
| Slc7a10 | 114518 | solute carrier family 7 member 10 | 0.0 | 5.7 | 0.008 |
| Homer1 | 29546 | homer scaffold protein 1 | 0.0 | 7.2 | 0.021 |
| Cnot6 | 287249 | CCR4-NOT transcription complex, subunit 6 | 0.0 | 5.1 | 0.012 |
| Azin1 | 58961 | antizyme inhibitor 1 | 0.0 | 6.5 | 0.044 |
| Jak2 | 24514 | Janus kinase 2 | 0.0 | 5.6 | 0.026 |
| Spcs2 | 293142 | signal peptidase complex subunit 2 | 0.0 | 7.3 | 0.045 |
| Kremen1 | 114107 | kringle containing transmembrane protein 1 | 0.0 | 6.5 | 0.042 |
| Nxph4 | 59316 | neurexophilin 4 | 0.0 | 7.2 | 0.049 |
| Dhx35 | 362260 | DEAH-box helicase 35 | 0.0 | 4.8 | 0.024 |
| Papss2 | 294103 | 3'-phosphoadenosine 5'-phosphosulfate synthase 2 | 0.0 | 3.9 | 0.004 |
| Rapgef5 | 362799 | Rap guanine nucleotide exchange factor 5 | 0.0 | 6.8 | 0.011 |
| Tspan13 | 366602 | tetraspanin 13 | 0.0 | 7.5 | 0.043 |
| Lrrn1 | 500280 | leucine rich repeat neuronal 1 | 0.0 | 5.6 | 0.046 |
| Zfp512b | 311721 | zinc finger protein 512B | 0.0 | 6.2 | 0.009 |
| Per3 | 78962 | period circadian regulator 3 | 0.0 | 5.5 | 0.016 |
| Slc35e1 | 498599 | solute carrier family 35, member E1 | 0.0 | 6.6 | 0.046 |
| Arih1 | 300756 | ariadne RBR E3 ubiquitin protein ligase 1 | 0.0 | 6.6 | 0.029 |
| Ets2 | 304063 | ETS proto-oncogene 2, transcription factor | 0.0 | 6.9 | 0.017 |
| Cc2d1b | 313478 | coiled-coil and C2 domain containing 1B | 0.0 | 5.4 | 0.049 |
| Cep95 | 287766 | centrosomal protein 95 | 0.0 | 4.0 | 0.002 |
| **Down-regulated DEGs (314 genes)** | | | | | |
| **Symbol** | **EntrezID** | **Description** | **Log2FC** | **logCPM** | **FDR (adjusted *p*-value)** |
| Car3 | 54232 | carbonic anhydrase 3 | -7.1 | 1.8 | 0.008 |
| Calcr | 116506 | calcitonin receptor | -4.9 | 1.2 | 0.046 |
| Cartpt | 29131 | CART prepropeptide | -3.3 | 3.3 | 0.001 |
| Cat | 24248 | catalase | -2.0 | 4.8 | 0.002 |
| Pde1a | 81529 | phosphodiesterase 1A | -2.0 | 3.2 | 0.027 |
| Nrp2 | 81527 | neuropilin 2 | -1.9 | 1.3 | 0.043 |
| Tnfrsf11b | 25341 | TNF receptor superfamily member 11B | -1.8 | 3.9 | 0.002 |
| Cyp2j10 | 313373 | cytochrome P450, family 2, subfamily j, polypeptide 10 | -1.8 | 2.9 | 0.008 |
| Mpeg1 | 64552 | macrophage expressed 1 | -1.7 | 6.1 | 0.010 |
| Cx3cr1 | 171056 | C-X3-C motif chemokine receptor 1 | -1.7 | 4.8 | 0.015 |
| Tlr2 | 310553 | toll-like receptor 2 | -1.7 | 2.4 | 0.019 |
| Atp2b4 | 29600 | ATPase plasma membrane Ca2+ transporting 4 | -1.7 | 4.4 | 0.028 |
| Creb5 | 500131 | cAMP responsive element binding protein 5 | -1.7 | 1.9 | 0.029 |
| Asb13 | 361268 | ankyrin repeat and SOCS box-containing 13 | -1.7 | 3.6 | 0.032 |
| Cyyr1 | 304138 | cysteine and tyrosine rich 1 | -1.7 | 4.4 | 0.036 |
| Pcdhga10 | 498849 | protocadherin gamma subfamily A, 10 | -1.7 | 3.7 | 0.039 |
| Gtf2a1 | 83830 | general transcription factor 2A subunit 1 | -1.7 | 3.1 | 0.041 |
| Mical1 | 294520 | microtubule associated monooxygenase, calponin and LIM domain containing 1 | -1.7 | 2.3 | 0.044 |
| Slc24a2 | 84550 | solute carrier family 24 member 2 | -1.6 | 8.2 | 0.002 |
| Fktn | 362520 | fukutin | -1.6 | 5.0 | 0.004 |
| Nfib | 29227 | nuclear factor I/B | -1.6 | 1.9 | 0.007 |
| Rab9a | 84589 | RAB9A, member RAS oncogene family | -1.6 | 4.0 | 0.008 |
| Cdh19 | 360835 | cadherin 19 | -1.6 | 1.4 | 0.026 |
| Hipk2 | 362342 | homeodomain interacting protein kinase 2 | -1.6 | 4.7 | 0.026 |
| Gabrb3 | 24922 | gamma-aminobutyric acid type A receptor subunit beta 3 | -1.6 | 6.0 | 0.031 |
| Prox1 | 305066 | prospero homeobox 1 | -1.6 | 2.4 | 0.039 |
| Elavl3 | 282824 | ELAV like RNA binding protein 3 | -1.5 | 6.1 | 0.006 |
| Kcna2 | 25468 | potassium voltage-gated channel subfamily A member 2 | -1.5 | 3.5 | 0.020 |
| Edn1 | 24323 | endothelin 1 | -1.5 | 2.1 | 0.026 |
| Bbx | 303970 | BBX high mobility group box domain containing | -1.5 | 2.8 | 0.031 |
| Kcnk9 | 84429 | potassium two pore domain channel subfamily K member 9 | -1.5 | 2.2 | 0.031 |
| Egfr | 24329 | epidermal growth factor receptor | -1.5 | 2.5 | 0.032 |
| Lifr | 81680 | LIF receptor subunit alpha | -1.5 | 2.8 | 0.046 |
| Gpr34 | 554353 | G protein-coupled receptor 34 | -1.5 | 3.9 | 0.049 |
| Caln1 | 363909 | calneuron 1 | -1.5 | 4.7 | 0.049 |
| Ch25h | 309527 | cholesterol 25-hydroxylase | -1.4 | 1.7 | 0.004 |
| RGD1563349 | 502727 | endosome-lysosome associated apoptosis and autophagy regulator family member 2 | -1.4 | 6.2 | 0.004 |
| Prkcd | 170538 | protein kinase C, delta | -1.4 | 4.5 | 0.005 |
| LOC100361083 | 100361083 | hypothetical | -1.4 | 2.4 | 0.010 |
| Klf7 | 363243 | Kruppel like factor 7 | -1.4 | 3.8 | 0.010 |
| Snap25 | 25012 | synaptosome associated protein 25 | -1.4 | 10.0 | 0.012 |
| Tie1 | 89806 | tyrosine kinase with immunoglobulin-like and EGF-like domains 1 | -1.4 | 5.4 | 0.015 |
| Exoc4 | 116654 | exocyst complex component 4 | -1.4 | 5.3 | 0.015 |
| Usp45 | 313098 | ubiquitin specific peptidase 45 | -1.4 | 3.5 | 0.015 |
| Camk2d | 24246 | calcium/calmodulin-dependent protein kinase II delta | -1.4 | 4.3 | 0.016 |
| Grk5 | 59075 | G protein-coupled receptor kinase 5 | -1.4 | 4.3 | 0.021 |
| Kpna3 | 361055 | karyopherin subunit alpha 3 | -1.4 | 5.0 | 0.021 |
| Map2 | 25595 | microtubule-associated protein 2 | -1.4 | 8.1 | 0.023 |
| Setd8 | 689820 | lysine methyltransferase 5A | -1.4 | 4.6 | 0.025 |
| Erbb3 | 29496 | erb-b2 receptor tyrosine kinase 3 | -1.4 | 5.1 | 0.025 |
| RGD1309104 | 289084 | similar to RIKEN cDNA 1700025G04 gene | -1.4 | 4.0 | 0.028 |
| Tgfbr1 | 29591 | transforming growth factor, beta receptor 1 | -1.4 | 4.9 | 0.029 |
| Abcc9 | 25560 | ATP binding cassette subfamily C member 9 | -1.4 | 4.3 | 0.029 |
| Nmnat2 | 289095 | nicotinamide nucleotide adenylyltransferase 2 | -1.4 | 4.9 | 0.033 |
| Ppp1r12b | 304813 | protein phosphatase 1, regulatory subunit 12B | -1.4 | 6.9 | 0.035 |
| Gpd2 | 25062 | glycerol-3-phosphate dehydrogenase 2 | -1.4 | 3.6 | 0.036 |
| Xiap | 63879 | X-linked inhibitor of apoptosis | -1.4 | 4.3 | 0.039 |
| St18 | 266680 | ST18 C2H2C-type zinc finger transcription factor | -1.4 | 3.0 | 0.039 |
| Slco2b1 | 140860 | solute carrier organic anion transporter family, member 2b1 | -1.4 | 5.7 | 0.040 |
| Opalin | 361757 | oligodendrocytic myelin paranodal and inner loop protein | -1.4 | 7.3 | 0.045 |
| Zfp46 | 298558 | zinc finger protein 46 | -1.4 | 5.7 | 0.046 |
| Pak3 | 29433 | p21 (RAC1) activated kinase 3 | -1.4 | 3.6 | 0.046 |
| Ptprt | 362263 | protein tyrosine phosphatase, receptor type, T | -1.4 | 5.4 | 0.050 |
| Arl4a | 29308 | ADP-ribosylation factor like GTPase 4A | -1.3 | 4.2 | 0.002 |
| P2ry1 | 25265 | purinergic receptor P2Y1 | -1.3 | 4.5 | 0.003 |
| Spon1 | 64456 | spondin 1 | -1.3 | 6.1 | 0.004 |
| Jag1 | 29146 | jagged canonical Notch ligand 1 | -1.3 | 4.0 | 0.008 |
| Slco1c1 | 84511 | solute carrier organic anion transporter family, member 1c1 | -1.3 | 6.8 | 0.008 |
| Zfp317 | 500950 | zinc finger protein 317 | -1.3 | 4.1 | 0.010 |
| Lypd1 | 360838 | Ly6/Plaur domain containing 1 | -1.3 | 6.6 | 0.010 |
| Ncam1 | 24586 | neural cell adhesion molecule 1 | -1.3 | 7.7 | 0.012 |
| St8sia3 | 25547 | ST8 alpha-N-acetyl-neuraminide alpha-2,8-sialyltransferase 3 | -1.3 | 5.7 | 0.014 |
| Oas1b | 246268 | 2-5 oligoadenylate synthetase 1B | -1.3 | 3.0 | 0.014 |
| Ptprg | 171357 | protein tyrosine phosphatase, receptor type, G | -1.3 | 4.8 | 0.015 |
| Htr2c | 25187 | 5-hydroxytryptamine receptor 2C | -1.3 | 7.5 | 0.016 |
| Pdcd4 | 64031 | programmed cell death 4 | -1.3 | 5.8 | 0.016 |
| Kcnq3 | 29682 | potassium voltage-gated channel subfamily Q member 3 | -1.3 | 6.0 | 0.016 |
| Tenm2 | 117242 | teneurin transmembrane protein 2 | -1.3 | 6.3 | 0.017 |
| Cdh2 | 83501 | cadherin 2 | -1.3 | 6.4 | 0.018 |
| Cited4 | 114491 | Cbp/p300-interacting transactivator, with Glu/Asp-rich carboxy-terminal domain, 4 | -1.3 | 4.3 | 0.020 |
| Prom1 | 60357 | prominin 1 | -1.3 | 4.9 | 0.021 |
| Slc6a6 | 29464 | solute carrier family 6 member 6 | -1.3 | 6.0 | 0.021 |
| Tmcc3 | 314751 | transmembrane and coiled-coil domain family 3 | -1.3 | 5.9 | 0.023 |
| Ppfia2 | 362876 | PTPRF interacting protein alpha 2 | -1.3 | 5.2 | 0.024 |
| Grin2a | 24409 | glutamate ionotropic receptor NMDA type subunit 2A | -1.3 | 3.8 | 0.025 |
| Optn | 246294 | optineurin | -1.3 | 5.2 | 0.026 |
| Sarnp | 362819 | SAP domain containing ribonucleoprotein | -1.3 | 5.2 | 0.027 |
| Sgms2 | 310849 | sphingomyelin synthase 2 | -1.3 | 1.4 | 0.027 |
| Gabra2 | 289606 | gamma-aminobutyric acid type A receptor subunit alpha 2 | -1.3 | 6.1 | 0.028 |
| Grid1 | 79219 | glutamate ionotropic receptor delta type subunit 1 | -1.3 | 6.0 | 0.030 |
| Sema4c | 301346 | semaphorin 4C | -1.3 | 4.9 | 0.031 |
| Slk | 54308 | STE20-like kinase | -1.3 | 5.8 | 0.031 |
| Arhgap5 | 299012 | Rho GTPase activating protein 5 | -1.3 | 5.1 | 0.033 |
| Grem2 | 289264 | gremlin 2, DAN family BMP antagonist | -1.3 | 4.6 | 0.033 |
| Gabbr2 | 83633 | gamma-aminobutyric acid type B receptor subunit 2 | -1.3 | 5.9 | 0.033 |
| Taok1 | 286993 | TAO kinase 1 | -1.3 | 4.7 | 0.034 |
| Trip11 | 314393 | thyroid hormone receptor interactor 11 | -1.3 | 4.5 | 0.035 |
| Dscaml1 | 315615 | DS cell adhesion molecule-like 1 | -1.3 | 5.0 | 0.035 |
| Scrn3 | 311731 | secernin 3 | -1.3 | 3.8 | 0.036 |
| Nr2c2 | 50659 | nuclear receptor subfamily 2, group C, member 2 | -1.3 | 5.0 | 0.036 |
| Cfh | 155012 | complement factor H | -1.3 | 4.8 | 0.037 |
| Klhl23 | 311114 | kelch-like family member 23 | -1.3 | 5.7 | 0.038 |
| Irf8 | 292060 | interferon regulatory factor 8 | -1.3 | 3.6 | 0.038 |
| Pex5l | 286937 | peroxisomal biogenesis factor 5-like | -1.3 | 5.1 | 0.039 |
| Bmpr2 | 140590 | bone morphogenetic protein receptor type 2 | -1.3 | 5.3 | 0.039 |
| Chn1 | 84030 | chimerin 1 | -1.3 | 9.5 | 0.039 |
| Ppm1e | 360593 | protein phosphatase, Mg2+/Mn2+ dependent, 1E | -1.3 | 3.9 | 0.043 |
| Cpd | 25306 | carboxypeptidase D | -1.3 | 5.9 | 0.044 |
| RGD1561849 | 500393 | vexin | -1.3 | 5.5 | 0.045 |
| Slc1a4 | 305540 | solute carrier family 1 member 4 | -1.3 | 5.0 | 0.046 |
| Adgrf5 | 245977 | adhesion G protein-coupled receptor F5 | -1.3 | 5.3 | 0.050 |
| Pcdh9 | 306091 | protocadherin 9 | -1.3 | 5.4 | 0.050 |
| Glg1 | 29476 | golgi glycoprotein 1 | -1.3 | 7.1 | 0.050 |
| Fgfrl1 | 360903 | fibroblast growth factor receptor-like 1 | -1.2 | 4.1 | 0.003 |
| Dpy19l3 | 308519 | dpy-19 like C-mannosyltransferase 3 | -1.2 | 8.1 | 0.003 |
| Rragd | 297960 | Ras-related GTP binding D | -1.2 | 5.4 | 0.004 |
| Strn | 29149 | striatin | -1.2 | 7.9 | 0.006 |
| Sat1 | 302642 | spermidine/spermine N1-acetyl transferase 1 | -1.2 | 6.8 | 0.007 |
| Sv2b | 117556 | synaptic vesicle glycoprotein 2b | -1.2 | 7.5 | 0.007 |
| Ptprj | 29645 | protein tyrosine phosphatase, receptor type, J | -1.2 | 6.7 | 0.007 |
| Camkk1 | 60341 | calcium/calmodulin-dependent protein kinase kinase 1 | -1.2 | 7.2 | 0.008 |
| Samd14 | 287637 | sterile alpha motif domain containing 14 | -1.2 | 5.1 | 0.010 |
| Cadps | 26989 | calcium dependent secretion activator | -1.2 | 5.7 | 0.011 |
| Kdr | 25589 | kinase insert domain receptor | -1.2 | 4.0 | 0.014 |
| Hpcal1 | 50871 | hippocalcin-like 1 | -1.2 | 5.9 | 0.015 |
| Mcam | 78967 | melanoma cell adhesion molecule | -1.2 | 5.9 | 0.015 |
| Pdgfrb | 24629 | platelet derived growth factor receptor beta | -1.2 | 5.4 | 0.015 |
| Rbpj | 679028 | recombination signal binding protein for immunoglobulin kappa J region | -1.2 | 4.9 | 0.016 |
| Slc24a3 | 85267 | solute carrier family 24 member 3 | -1.2 | 6.3 | 0.017 |
| Gpcpd1 | 362219 | glycerophosphocholine phosphodiesterase 1 | -1.2 | 6.4 | 0.022 |
| Smcr8 | 497918 | SMCR8-C9orf72 complex subunit | -1.2 | 4.3 | 0.022 |
| Gnai1 | 25686 | G protein subunit alpha i1 | -1.2 | 6.5 | 0.023 |
| Man1c1 | 362625 | mannosidase, alpha, class 1C, member 1 | -1.2 | 5.9 | 0.024 |
| Cacng2 | 84347 | calcium voltage-gated channel auxiliary subunit gamma 2 | -1.2 | 3.9 | 0.024 |
| Astn2 | 100361323 | astrotactin 2 | -1.2 | 4.2 | 0.025 |
| Cdc73 | 304832 | cell division cycle 73 | -1.2 | 3.5 | 0.025 |
| Plcb4 | 25031 | phospholipase C, beta 4 | -1.2 | 4.6 | 0.025 |
| Mpped1 | 362971 | metallophosphoesterase domain containing 1 | -1.2 | 7.1 | 0.026 |
| Lap3 | 289668 | leucine aminopeptidase 3 | -1.2 | 6.4 | 0.026 |
| Sema5a | 310207 | semaphorin 5A | -1.2 | 5.6 | 0.026 |
| Rasgrp1 | 29434 | RAS guanyl releasing protein 1 | -1.2 | 8.8 | 0.026 |
| Nr3c1 | 24413 | nuclear receptor subfamily 3, group C, member 1 | -1.2 | 6.7 | 0.026 |
| Egfl7 | 245963 | EGF-like-domain, multiple 7 | -1.2 | 5.1 | 0.027 |
| Rbm3 | 114488 | RNA binding motif (RNP1, RRM) protein 3 | -1.2 | 6.4 | 0.027 |
| Nrxn3 | 116508 | neurexin 3 | -1.2 | 6.9 | 0.027 |
| Adamts4 | 66015 | ADAM metallopeptidase with thrombospondin type 1 motif, 4 | -1.2 | 5.7 | 0.027 |
| Baz2b | 317627 | bromodomain adjacent to zinc finger domain, 2B | -1.2 | 4.8 | 0.027 |
| Ppp2r2b | 60660 | protein phosphatase 2, regulatory subunit B, beta | -1.2 | 7.1 | 0.028 |
| Lgals9 | 25476 | galectin 9 | -1.2 | 4.3 | 0.029 |
| Tulp4 | 499016 | TUB like protein 4 | -1.2 | 6.1 | 0.030 |
| Prkch | 81749 | protein kinase C, eta | -1.2 | 5.7 | 0.030 |
| Extl3 | 56819 | exostosin-like glycosyltransferase 3 | -1.2 | 5.7 | 0.030 |
| Zdhhc21 | 298184 | zinc finger DHHC-type palmitoyltransferase 21 | -1.2 | 3.7 | 0.030 |
| Scn2a | 24766 | sodium voltage-gated channel alpha subunit 2 | -1.2 | 8.0 | 0.031 |
| March7 | 311059 | membrane associated ring-CH-type finger 7 | -1.2 | 4.8 | 0.031 |
| Fnip2 | 310538 | folliculin interacting protein 2 | -1.2 | 4.1 | 0.032 |
| Usp12 | 360763 | ubiquitin specific peptidase 12 | -1.2 | 4.4 | 0.032 |
| Cacnb4 | 58942 | calcium voltage-gated channel auxiliary subunit beta 4 | -1.2 | 5.8 | 0.032 |
| Tgfbr2 | 81810 | transforming growth factor, beta receptor 2 | -1.2 | 4.3 | 0.033 |
| Grb14 | 58844 | growth factor receptor bound protein 14 | -1.2 | 4.3 | 0.033 |
| Ddx6 | 500988 | DEAD-box helicase 6 | -1.2 | 5.4 | 0.033 |
| Ostm1 | 499474 | osteoclastogenesis associated transmembrane protein 1 | -1.2 | 5.9 | 0.034 |
| Parl | 287979 | presenilin associated, rhomboid-like | -1.2 | 5.1 | 0.034 |
| Rasa3 | 29372 | RAS p21 protein activator 3 | -1.2 | 4.9 | 0.035 |
| Yipf6 | 363476 | Yip1 domain family, member 6 | -1.2 | 5.0 | 0.035 |
| Stam2 | 311030 | signal transducing adaptor molecule 2 | -1.2 | 3.5 | 0.035 |
| Cmtm4 | 498902 | CKLF-like MARVEL transmembrane domain containing 4 | -1.2 | 3.6 | 0.036 |
| Dars | 116483 | aspartyl-tRNA synthetase 1 | -1.2 | 5.2 | 0.036 |
| Fam81a | 315789 | family with sequence similarity 81, member A | -1.2 | 4.7 | 0.036 |
| Kif21b | 289397 | kinesin family member 21B | -1.2 | 5.3 | 0.037 |
| Tmem18 | 362722 | transmembrane protein 18 | -1.2 | 3.7 | 0.037 |
| Fnbp1 | 192348 | formin binding protein 1 | -1.2 | 6.3 | 0.038 |
| Mon2 | 314894 | MON2 homolog, regulator of endosome-to-Golgi trafficking | -1.2 | 5.7 | 0.038 |
| Med1 | 497991 | mediator complex subunit 1 | -1.2 | 5.2 | 0.038 |
| Gpr37 | 117549 | G protein-coupled receptor 37 | -1.2 | 7.6 | 0.039 |
| Sgpl1 | 286896 | sphingosine-1-phosphate lyase 1 | -1.2 | 4.4 | 0.039 |
| Fzd10 | 363913 | frizzled class receptor 10 | -1.2 | 5.3 | 0.039 |
| Sptlc2 | 366697 | serine palmitoyltransferase, long chain base subunit 2 | -1.2 | 5.2 | 0.039 |
| Rnf31 | 364386 | ring finger protein 31 | -1.2 | 5.0 | 0.039 |
| Klhl9 | 313348 | kelch-like family member 9 | -1.2 | 6.7 | 0.041 |
| Kif13b | 305967 | kinesin family member 13B | -1.2 | 5.1 | 0.044 |
| Htr6 | 64354 | 5-hydroxytryptamine receptor 6 | -1.2 | 5.0 | 0.044 |
| Gpc3 | 25236 | glypican 3 | -1.2 | 2.7 | 0.044 |
| Sulf2 | 311642 | sulfatase 2 | -1.2 | 6.8 | 0.045 |
| Prkcb | 25023 | protein kinase C, beta | -1.2 | 8.8 | 0.045 |
| Adck5 | 362943 | aarF domain containing kinase 5 | -1.2 | 5.1 | 0.045 |
| Opa1 | 171116 | OPA1, mitochondrial dynamin like GTPase | -1.2 | 6.3 | 0.046 |
| Pkia | 114906 | cAMP-dependent protein kinase inhibitor alpha | -1.2 | 7.0 | 0.047 |
| Tmem229a | 685756 | transmembrane protein 229A | -1.2 | 6.9 | 0.047 |
| Gabpa | 363735 | GA binding protein transcription factor subunit alpha | -1.2 | 4.8 | 0.047 |
| Slc4a4 | 84484 | solute carrier family 4 member 4 | -1.2 | 6.0 | 0.048 |
| Mtmr12 | 310155 | myotubularin related protein 12 | -1.2 | 4.1 | 0.049 |
| Sms | 363469 | spermine synthase | -1.2 | 5.8 | 0.049 |
| Ubox5 | 296161 | U-box domain containing 5 | -1.2 | 4.5 | 0.050 |
| RGD1310587 | 360894 | similar to hypothetical protein FLJ14146 | -1.2 | 4.9 | 0.050 |
| Mef2a | 309957 | myocyte enhancer factor 2a | -1.2 | 6.5 | 0.050 |
| Pcm1 | 81740 | pericentriolar material 1 | -1.2 | 6.4 | 0.050 |
| Srsf5 | 29667 | serine and arginine rich splicing factor 5 | -1.1 | 7.7 | 0.000 |
| Plxnb3 | 363517 | plexin B3 | -1.1 | 5.3 | 0.001 |
| Slc12a9 | 171443 | solute carrier family 12, member 9 | -1.1 | 4.8 | 0.001 |
| Slc9a1 | 24782 | solute carrier family 9 member A1 | -1.1 | 6.8 | 0.003 |
| Megf10 | 291445 | multiple EGF-like domains 10 | -1.1 | 4.4 | 0.003 |
| Nfxl1 | 289595 | nuclear transcription factor, X-box binding-like 1 | -1.1 | 4.3 | 0.004 |
| Cirbp | 81825 | cold inducible RNA binding protein | -1.1 | 6.4 | 0.005 |
| Mmp14 | 81707 | matrix metallopeptidase 14 | -1.1 | 3.3 | 0.007 |
| Arhgap27 | 303583 | Rho GTPase activating protein 27 | -1.1 | 5.2 | 0.007 |
| Sesn1 | 294518 | sestrin 1 | -1.1 | 6.3 | 0.007 |
| Grm3 | 24416 | glutamate metabotropic receptor 3 | -1.1 | 6.3 | 0.008 |
| Fam160b2 | 306015 | FHF complex subunit HOOK interacting protein 2B | -1.1 | 6.6 | 0.011 |
| Insig1 | 64194 | insulin induced gene 1 | -1.1 | 6.0 | 0.011 |
| Slc38a1 | 170567 | solute carrier family 38, member 1 | -1.1 | 6.3 | 0.011 |
| Thrb | 24831 | thyroid hormone receptor beta | -1.1 | 6.3 | 0.015 |
| Pbx1 | 304947 | PBX homeobox 1 | -1.1 | 5.3 | 0.015 |
| Mbnl1 | 282635 | muscleblind-like splicing regulator 1 | -1.1 | 4.3 | 0.015 |
| Slc16a6 | 303772 | solute carrier family 16, member 6 | -1.1 | 3.9 | 0.015 |
| Mus81 | 293678 | MUS81 structure-specific endonuclease subunit | -1.1 | 4.2 | 0.016 |
| Rft1 | 290552 | RFT1 homolog | -1.1 | 4.1 | 0.019 |
| Camk1g | 171358 | calcium/calmodulin-dependent protein kinase IG | -1.1 | 4.2 | 0.019 |
| Myt1l | 116668 | myelin transcription factor 1-like | -1.1 | 7.1 | 0.019 |
| Fam136a | 297415 | family with sequence similarity 136, member A | -1.1 | 4.7 | 0.021 |
| Cggbp1 | 288353 | CGG triplet repeat binding protein 1 | -1.1 | 5.9 | 0.022 |
| Ydjc | 287938 | YdjC chitooligosaccharide deacetylase homolog | -1.1 | 5.8 | 0.022 |
| Lrrn3 | 81514 | leucine rich repeat neuronal 3 | -1.1 | 5.4 | 0.022 |
| Slc7a1 | 25648 | solute carrier family 7 member 1 | -1.1 | 5.9 | 0.023 |
| Appl2 | 362860 | adaptor protein, phosphotyrosine interacting with PH domain and leucine zipper 2 | -1.1 | 5.6 | 0.023 |
| Amdhd2 | 302972 | amidohydrolase domain containing 2 | -1.1 | 4.0 | 0.025 |
| Ppm1k | 312381 | protein phosphatase, Mg2+/Mn2+ dependent, 1K | -1.1 | 5.5 | 0.025 |
| Sugp1 | 290666 | SURP and G patch domain containing 1 | -1.1 | 6.2 | 0.025 |
| Marcks | 25603 | myristoylated alanine rich protein kinase C substrate | -1.1 | 8.0 | 0.026 |
| Zfp148 | 58820 | zinc finger protein 148 | -1.1 | 5.3 | 0.026 |
| Cdc42bpa | 114116 | CDC42 binding protein kinase alpha | -1.1 | 6.7 | 0.026 |
| Crebzf | 293112 | CREB/ATF bZIP transcription factor | -1.1 | 6.3 | 0.026 |
| Usp32 | 303394 | ubiquitin specific peptidase 32 | -1.1 | 6.8 | 0.029 |
| Srebf1 | 78968 | sterol regulatory element binding transcription factor 1 | -1.1 | 6.9 | 0.030 |
| B3galt1 | 366064 | Beta-1,3-galactosyltransferase 1 | -1.1 | 4.2 | 0.030 |
| Pdzd8 | 308000 | PDZ domain containing 8 | -1.1 | 6.7 | 0.031 |
| Slc29a3 | 353307 | solute carrier family 29 member 3 | -1.1 | 4.4 | 0.031 |
| Rabep2 | 80754 | rabaptin, RAB GTPase binding effector protein 2 | -1.1 | 4.6 | 0.032 |
| Fbxw9 | 288921 | F-box and WD repeat domain containing 9 | -1.1 | 4.2 | 0.032 |
| Ncan | 58982 | neurocan | -1.1 | 7.0 | 0.032 |
| Mppe1 | 361344 | metallophosphoesterase 1 | -1.1 | 5.1 | 0.033 |
| Selt | 365802 | selenoprotein T | -1.1 | 7.2 | 0.033 |
| Mapk10 | 25272 | mitogen activated protein kinase 10 | -1.1 | 6.3 | 0.034 |
| Rybp | 312603 | RING1 and YY1 binding protein | -1.1 | 6.4 | 0.034 |
| Slc20a2 | 29502 | solute carrier family 20 member 2 | -1.1 | 6.1 | 0.035 |
| Mb21d2 | 498100 | Mab-21 domain containing 2 | -1.1 | 5.7 | 0.037 |
| Ciart | 365871 | circadian associated repressor of transcription | -1.1 | 4.5 | 0.038 |
| Phldb1 | 171434 | pleckstrin homology-like domain, family B, member 1 | -1.1 | 7.6 | 0.039 |
| Trim2 | 361970 | tripartite motif-containing 2 | -1.1 | 7.5 | 0.039 |
| Hs3st1 | 84406 | heparan sulfate-glucosamine 3-sulfotransferase 1 | -1.1 | 5.0 | 0.040 |
| Rffl | 282844 | ring finger and FYVE-like domain containing E3 ubiquitin protein ligase | -1.1 | 5.1 | 0.041 |
| Slitrk1 | 306147 | SLIT and NTRK-like family, member 1 | -1.1 | 5.0 | 0.042 |
| Coasy | 287711 | Coenzyme A synthase | -1.1 | 5.2 | 0.042 |
| Shroom3 | 305230 | shroom family member 3 | -1.1 | 1.6 | 0.042 |
| Lrfn5 | 314164 | leucine rich repeat and fibronectin type III domain containing 5 | -1.1 | 5.1 | 0.043 |
| Dnajc16 | 362652 | DnaJ heat shock protein family (Hsp40) member C16 | -1.1 | 5.4 | 0.043 |
| Astn1 | 304900 | astrotactin 1 | -1.1 | 7.6 | 0.043 |
| Rasip1 | 292912 | Ras interacting protein 1 | -1.1 | 4.8 | 0.044 |
| Lrrc4c | 311236 | leucine rich repeat containing 4C | -1.1 | 6.6 | 0.047 |
| Abca2 | 79248 | ATP binding cassette subfamily A member 2 | -1.1 | 9.0 | 0.047 |
| Fnbp1l | 310839 | formin binding protein 1-like | -1.1 | 5.6 | 0.047 |
| Map4k2 | 293694 | mitogen activated protein kinase kinase kinase kinase 2 | -1.1 | 5.9 | 0.048 |
| Fzd3 | 266715 | frizzled class receptor 3 | -1.1 | 3.7 | 0.049 |
| Dusp15 | 362238 | dual specificity phosphatase 15 | -1.1 | 5.9 | 0.049 |
| Xpot | 314879 | exportin for tRNA | -1.1 | 5.7 | 0.049 |
| Ptprz1 | 25613 | protein tyrosine phosphatase, receptor type Z, polypeptide 1 | -1.1 | 7.6 | 0.049 |
| Pfkl | 25741 | phosphofructokinase, liver type | -1.1 | 7.6 | 0.049 |
| Chst11 | 314694 | carbohydrate sulfotransferase 11 | -1.1 | 5.3 | 0.049 |
| Pnp | 290029 | purine nucleoside phosphorylase | -1.1 | 7.1 | 0.050 |
| Atp2b3 | 29599 | ATPase, Ca++ transporting, plasma membrane 3 | -1.1 | 6.4 | 0.050 |
| Rprd1a | 291736 | regulation of nuclear pre-mRNA domain containing 1A | -1.1 | 6.9 | 0.050 |
| Epha4 | 316539 | Eph receptor A4 | -1.1 | 8.1 | 0.050 |
| Hnrnpdl | 305178 | heterogeneous nuclear ribonucleoprotein D-like | -1.0 | 7.2 | 0.003 |
| Fus | 317385 | FUS RNA binding protein | -1.0 | 8.4 | 0.007 |
| Mthfd1l | 361472 | methylenetetrahydrofolate dehydrogenase (NADP+ dependent) 1-like | -1.0 | 4.3 | 0.008 |
| Dbp | 24309 | D-box binding PAR bZIP transcription factor | -1.0 | 9.4 | 0.011 |
| Csnk1g3 | 64823 | casein kinase 1, gamma 3 | -1.0 | 6.0 | 0.016 |
| Reck | 313488 | reversion-inducing-cysteine-rich protein with kazal motifs | -1.0 | 4.8 | 0.016 |
| Prrt4 | 500059 | proline-rich transmembrane protein 4 | -1.0 | 4.7 | 0.018 |
| Anxa6 | 79125 | annexin A6 | -1.0 | 7.1 | 0.021 |
| Map9 | 310544 | microtubule-associated protein 9 | -1.0 | 4.9 | 0.021 |
| Tiam1 | 304109 | TIAM Rac1 associated GEF 1 | -1.0 | 6.6 | 0.022 |
| Pstk | 361661 | phosphoseryl-tRNA kinase | -1.0 | 4.4 | 0.023 |
| Pik3ip1 | 305472 | phosphoinositide-3-kinase interacting protein 1 | -1.0 | 6.2 | 0.023 |
| Dffb | 84359 | DNA fragmentation factor subunit beta | -1.0 | 2.9 | 0.024 |
| Vopp1 | 362374 | VOPP1 WW domain binding protein | -1.0 | 7.1 | 0.024 |
| Scn1a | 81574 | sodium voltage-gated channel alpha subunit 1 | -1.0 | 4.7 | 0.026 |
| Smad3 | 25631 | SMAD family member 3 | -1.0 | 7.2 | 0.026 |
| Numa1 | 308870 | nuclear mitotic apparatus protein 1 | -1.0 | 7.1 | 0.026 |
| Zfyve28 | 305454 | zinc finger FYVE-type containing 28 | -1.0 | 6.9 | 0.027 |
| Brinp1 | 140610 | BMP/retinoic acid inducible neural specific 1 | -1.0 | 6.8 | 0.027 |
| Cyb5r3 | 25035 | cytochrome b5 reductase 3 | -1.0 | 6.3 | 0.027 |
| Grin2b | 24410 | glutamate ionotropic receptor NMDA type subunit 2B | -1.0 | 4.3 | 0.027 |
| Lgi3 | 306013 | leucine-rich repeat LGI family, member 3 | -1.0 | 6.7 | 0.027 |
| Mknk1 | 500526 | MAPK interacting serine/threonine kinase 1 | -1.0 | 4.6 | 0.030 |
| Abhd11 | 360831 | abhydrolase domain containing 11 | -1.0 | 4.0 | 0.032 |
| Tmem57 | 313618 | macoilin 1 | -1.0 | 6.1 | 0.032 |
| Cacng3 | 140724 | calcium voltage-gated channel auxiliary subunit gamma 3 | -1.0 | 6.6 | 0.033 |
| Acss2 | 311569 | acyl-CoA synthetase short-chain family member 2 | -1.0 | 5.7 | 0.033 |
| Fah | 29383 | fumarylacetoacetate hydrolase | -1.0 | 4.6 | 0.033 |
| Eif4g3 | 298573 | eukaryotic translation initiation factor 4 gamma, 3 | -1.0 | 7.5 | 0.033 |
| RGD1565616 | 499891 | dynein axonemal assembly factor 9 | -1.0 | 7.0 | 0.033 |
| Lmo1 | 245979 | LIM domain only 1 | -1.0 | 5.1 | 0.033 |
| Kbtbd8 | 500262 | kelch repeat and BTB domain containing 8 | -1.0 | 2.7 | 0.034 |
| Sos1 | 313845 | SOS Ras/Rac guanine nucleotide exchange factor 1 | -1.0 | 5.7 | 0.036 |
| Celf6 | 300758 | CUGBP, Elav-like family member 6 | -1.0 | 5.0 | 0.038 |
| RGD1310352 | 303122 | similar to HTGN29 protein; keratinocytes associated transmembrane protein 2 | -1.0 | 6.8 | 0.038 |
| Hip1r | 81917 | huntingtin interacting protein 1 related | -1.0 | 7.0 | 0.040 |
| Arhgap25 | 500246 | Rho GTPase activating protein 25 | -1.0 | 4.3 | 0.043 |
| Sorcs2 | 305438 | sortilin-related VPS10 domain containing receptor 2 | -1.0 | 7.2 | 0.044 |
| Ogdhl | 290566 | oxoglutarate dehydrogenase L | -1.0 | 6.5 | 0.044 |
| Cpsf1 | 366952 | cleavage and polyadenylation specific factor 1 | -1.0 | 6.1 | 0.045 |
| Kif3c | 85248 | kinesin family member 3C | -1.0 | 7.1 | 0.047 |
| Sh3rf1 | 306417 | SH3 domain containing ring finger 1 | -1.0 | 5.0 | 0.048 |
